# Specific inflammatory osteoclast precursors induced during chronic inflammation give rise to highly active osteoclasts associated with inflammatory bone loss

**DOI:** 10.1038/s41413-022-00206-z

**Published:** 2022-04-08

**Authors:** Yaron Meirow, Milena Jovanovic, Yuval Zur, Juliana Habib, Daniele Filippo Colombo, Nira Twaik, Hadas Ashkenazi-Preiser, Kerem Ben-Meir, Ivan Mikula, Or Reuven, Guy Kariv, Leonor Daniel, Saja Baraghithy, Yehuda Klein, Jeroen Krijgsveld, Noam Levaot, Michal Baniyash

**Affiliations:** 1 The Concern Foundation Laboratories at The Lautenberg Center for Immunology and Cancer Research, Israel-Canada Medical Research Institute, Faculty of Medicine, The Hebrew University, Jerusalem, Israel; 2 Department of Physiology and Cell Biology, Ben-Gurion University of the Negev, Beer-Sheva, Israel; 3 Division of Proteomics of Stem Cells and Cancer, German Cancer Research Center (DKFZ), Heidelberg, Germany; 4 Institute for Drug Research, Faculty of Medicine, The Hebrew University, Jerusalem, Israel; 5 Hebrew University-Hadassah School of Dental Medicine, Jerusalem, Israel; 6 Heidelberg University, Medical Faculty, Heidelberg, Germany

**Keywords:** Bone, Pathogenesis

## Abstract

Elevated osteoclast (OC) activity is a major contributor to inflammatory bone loss (IBL) during chronic inflammatory diseases. However, the specific OC precursors (OCPs) responding to inflammatory cues and the underlying mechanisms leading to IBL are poorly understood. We identified two distinct OCP subsets: Ly6C^hi^CD11b^hi^ inflammatory OCPs (iOCPs) induced during chronic inflammation, and homeostatic Ly6C^hi^CD11b^lo^ OCPs (hOCPs) which remained unchanged. Functional and proteomic characterization revealed that while iOCPs were rare and displayed low osteoclastogenic potential under normal conditions, they expanded during chronic inflammation and generated OCs with enhanced activity. In contrast, hOCPs were abundant and manifested high osteoclastogenic potential under normal conditions but generated OCs with low activity and were unresponsive to the inflammatory environment. Osteoclasts derived from iOCPs expressed higher levels of resorptive and metabolic proteins than those generated from hOCPs, highlighting that different osteoclast populations are formed by distinct precursors. We further identified the TNF-α and S100A8/A9 proteins as key regulators that control the iOCP response during chronic inflammation. Furthermore, we demonstrated that the response of iOCPs but not that of hOCPs was abrogated in *tnf-α*^−/−^ mice, in correlation with attenuated IBL. Our findings suggest a central role for iOCPs in IBL induction. iOCPs can serve as potential biomarkers for IBL detection and possibly as new therapeutic targets to combat IBL in a wide range of inflammatory conditions.

## Introduction

Inflammatory bone loss (IBL) occurs in a myriad of chronic inflammatory conditions. It manifests when the site of inflammation is adjacent to the bones, as in gingivitis^1^, peri-implantitis^2,3^, rheumatoid arthritis^4^, multiple myeloma^5^ and bone marrow (BM) metastases^6^. Systemic IBL has also been observed in unrelated bone diseases, such as low-grade inflammation in menopause^7^ and idiopathic inflammatory bowel disease (IBD)^8–10^. IBL and the role of osteoclasts (OCs) in its development are well known^11^; however, the regulatory mechanisms controlling specific OC precursor (OCP) responses that result in enhanced OC activity during chronic inflammation are still undetermined.

Chronic inflammation arises in a wide range of diseases when immunogenic stimuli persist^12^ and results in myelopoiesis alterations and the accumulation and differentiation arrest of heterogeneous immature myeloid cells, which comprise a major population termed myeloid-derived suppressor cells (MDSCs). MDSCs are found in the BM and periphery of chronically inflamed mice and humans and consist of both polymorphonuclear (PMN-MDSCs) and monocytic (M-MDSCs) cells, which both exert immunosuppressive activity during chronic inflammation^13–15^.

Various subsets of BM monocytes were reported to differentiate into OCs and were termed OCPs accordingly. These include Ly6C^hi^CD11b^lo^ OCPs^16^, Gr1^+^CD11b^+^ MDSCs^5,6,17^ and other populations defined by the general markers CD115, CD117 and CD11b^11^. All the abovementioned populations are heterogeneous and consist of monocytes in various stages of differentiation, such as the early Ly6C^hi^CD11b^lo^CD117^+^ committed monocyte precursors (cMoP), the later stage Ly6C^hi^CD11b^+^CD117^-^ differentiating monocytes (Ly6C^hi^ monocytes) and the further differentiated Ly6C^-^ monocytes^18,19^. Moreover, many of the previously reported OCP populations exhibit overlapping marker expression, for example, monocytic MDSCs and Ly6C^hi^ monocytes are both defined by Ly6C and CD11b expression. This emerging OCP heterogeneity presents a challenge in the study of the OCP response during chronic inflammation. Most importantly, it is unclear whether a specific OCP subset produces OCs with elevated activity or whether a general osteoclastogenic response is induced during chronic inflammation.

The proinflammatory cytokine tumor necrosis factor-α (TNF-α) was shown to have a major role as a master regulator of myeloid differentiation under both homeostatic and chronic inflammatory conditions. Physiological levels of TNF-α were shown to be essential for the differentiation of cMoPs into Ly6C^hi^ monocytes and further into Ly6C^-^ monocytes^19^. In contrast, we have previously shown that high levels of TNF-α induce the accumulation of MDSCs and prevent their differentiation into mature antigen-presenting cells (APCs) during chronic inflammation^20^. Excess TNF-α was also shown to induce arthritis and IBL in mice^21^, demonstrating its involvement in OC development in inflammatory settings.

In this report, we identify and characterize two functionally and molecularly distinct OCP subpopulations within the Ly6C^hi^ monocytic population that respond differently to chronic inflammation: Ly6C^hi^CD11b^hi^ inflammatory OCPs (iOCPs) and Ly6C^hi^CD11b^lo^ homeostatic OCPs (hOCPs). Among these cell types, only iOCPs responded to inflammatory settings via a TNF-α and S100A8/A9 protein loop by producing OCs with enhanced activity, while hOCPs were homeostatic and produced OCs with low activity. Moreover, we showed that only the iOCP response, not the hOCP response, correlated with IBL, suggesting that iOCPs may play a central role in bone loss induction during chronic inflammation.

## Results

### Chronic inflammation augments OC activity and differentially expands Ly6C^hi^ monocytic subpopulations

**Fig. 1:**
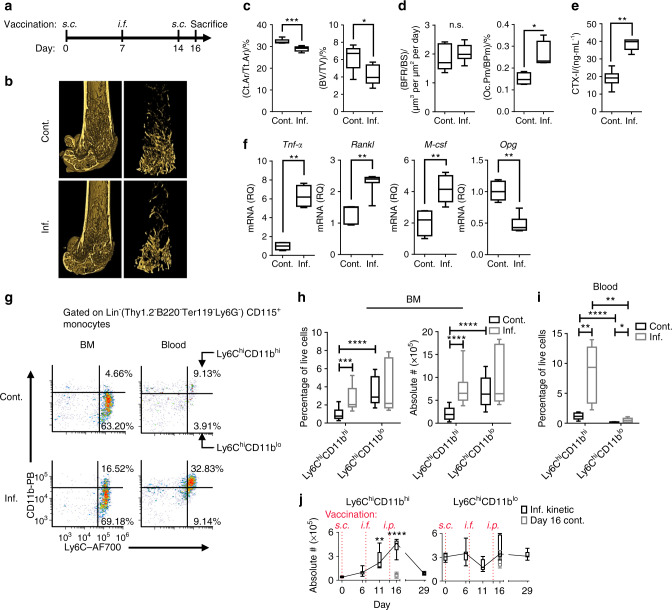
Chronic inflammation augments OC activity and expands Ly6Chi monocyte populations. **a** Time course of the vaccination model (see the Methods section for details). **b** Representative 3D modeling from femoral microCT scans of control (cont.) and inflamed (inf.) mice. All microCT scans were performed on the right femur. **c** MicroCT evaluation of the cortical bone cross-sectional area fraction (Ct.Ar/Tt.Ar) and trabecular bone volume/total volume (BV/TV). **d** Histomorphometric indices: bone formation rate/bone surface (BFR/BS) and fraction of OCs occupying the cortical perimeter (Oc.Pm/BPm). The full microCT and histomorphometric results are shown in Table S1. **e** Serum collagen type 1 cross-linked C-telopeptide (CTX-I) levels. **f** Relative quantities (RQs) of *Tnf-α*, *Rankl*, *M-csf* and *Opg* transcripts in RNA extracted from the proximal half of the right tibia. **a**–**f** Control *n* = 7, inflamed *n* = 7; representative results for three independent experiments (Mann–Whitney test and Holm multiplicity correction). **g** Flow cytometric analysis of leukocytes isolated from the BM and blood of control and inflamed mice. The plots are gated on Lin^-^(Thy1^.^2^-^B220^-^Ter119^-^Ly6G^-^)CD115^+^ monocytes and depict the Ly6C^hi^CD11b^hi^ and Ly6C^hi^CD11b^lo^ subsets. **h** Ly6C^hi^CD11b^hi^ and Ly6C^hi^CD11b^lo^ cell frequencies and absolute numbers in the BM of control and inflamed mice [absolute number in BM extracted from 1 leg (femur + tibia)]. **i** Frequency in the blood. Representative results for three independent experiments are presented. Control *n* = 15, inflamed *n* = 15 (Mann–Whitney test and Holm multiplicity correction). **j** Kinetics of Ly6C^hi^CD11b^hi^ and Ly6C^hi^CD11b^lo^ cell accumulation in the BM during the vaccination protocol. Inflamed *n* = 7 for each time point, Day 16 control *n* = 6 (Kruskal–Wallis and Dunn post-test all compared to Day 0). Line: median, box: 25^th^–75^th^ percentile, whiskers: range. *P < 0.05, **P < 0.01, ***P < 0.00 1, ****P < 0.000 1. (*s.c*. – subcutaneous, *i.f*. – intrafootpad, *i.p*. – intraperitoneal)

To characterize the skeletal response during chronic inflammation, we used a mouse model of chronic inflammation, mimicking conditions common to a wide range of chronic inflammatory diseases^15,22^. Briefly, mice were repeatedly vaccinated with heat-killed bacteria (Fig. 1a and the Methods section), generating chronic inflammatory conditions associated with dysregulated myelopoiesis reflected by the expansion and accumulation of the general MDSC population (Fig. S1a, b), including the monocytic (Fig. S1c, d) and PMN-MDSC subpopulations (Fig. S1e, f). This was accompanied by the downregulation of CD247 (Fig. S1g, h), indicating T cell dysfunction, as was previously described^13,14,20^.

Femoral microCT scans (Fig. 1b) showed significant decreases in cortical and trabecular bone mass (cross-sectional cortical area/total area (Ct.Ar/Tt.Ar) and trabecular bone volume/total volume (BV/TV), respectively) in the inflamed *vs*. control mice (Fig. 1c). Next, we measured in vivo indices of bone formation and resorption. The induced inflammation did not affect the bone formation rate/bone surface parameter but increased the fraction of cortical bone occupied by OCs (cross-sectional OC perimeter/bone perimeter (Oc.Pm/BPm)) (Fig. 1d), indicating enhanced bone resorption (see Table S1 for all microCT and histomorphometric analyses). The total OC activity in vivo was found to be elevated, as measured by increased serous collagen type 1 cross-linked C-telopeptide (CTX-I) (Fig. 1e). We found enhanced expression of *Tnf-α*, receptor activator of nuclear factor κB (RANK) ligand (*Rankl*) and macrophage colony stimulating factor (*M-csf*) transcripts in the proximal tibiae of inflamed mice, along with a decrease in the expression of the negative RANKL regulator osteoprotegerin (*Opg*) (Fig. 1f). These findings demonstrate favorable conditions for OC formation and activity and indicate that IBL is an immune cell-based phenomenon, as it is driven by enhanced OC activity in the context of normal osteoblast activity.

To characterize the cellular changes underlying the observed bone loss during chronic inflammation, we focused on Ly6C^hi^ monocytes as the major source of OCPs. Two subpopulations of lineage-negative (Lin^-^: Thy1.2^-^B220^-^Ter119^-^Ly6G^-^) Ly6C^hi^ monocytes with low or high CD11b expression were observed in the BM and blood (Fig. 1g). Ly6C^hi^CD11b^lo^ cells represented the dominant population in the BM of control mice and were not affected during chronic inflammation. In contrast, Lin^-^Ly6C^hi^CD11b^hi^ cells represented a minor population under normal conditions but were significantly expanded in the BM and blood under chronic inflammation, both in relative and absolute numbers (Fig. 1h, i). Ly6C^hi^CD11b^hi^ cells gradually accumulated in the BM along with the development of chronic inflammation and dissipated upon recovery (Day 29) (Fig. 1j, left panel). Ly6C^hi^CD11b^lo^ cells remained at steady numbers throughout the entire duration of the experiments (Fig. 1j, right panel). Thus, it was suspected that Ly6C^hi^CD11b^hi^ cells represent an inflammatory population, while Ly6C^hi^CD11b^lo^ cells are homeostatic, as the former expanded during chronic inflammation and the latter did not.

To test whether the accumulation of Ly6C^hi^CD11b^hi^ cells but not Ly6C^hi^CD11b^lo^ cells during chronic inflammation is a general phenomenon, we tested another mouse model system of dextran sodium sulfate (DSS)-induced chronic inflammatory bowel disease (IBD), which resembles human disease. IBD mice displayed colonic inflammation (Fig. S2a) associated with weight loss (Fig. S2b) and accumulation of MDSCs in the BM and spleen (Fig. S2c), which was accompanied by CD247 downregulation in splenic T cells with no change in CD3e expression (Fig. S2d), indicating chronic inflammation and T cell suppression. IBD mice displayed loss of trabecular bone (Fig. S2e), and similar to the results for the vaccine-based model, only Ly6C^hi^CD11b^hi^ cells responded to the induced inflammation and accumulated in the BM of inflamed mice (Fig. S2f). These results show that Ly6C^hi^CD11b^hi^ cells but not Ly6C^hi^CD11b^lo^ cells accumulate during chronic inflammation in the two tested models. This differential responses of the subpopulations of Ly6C^hi^ monocytes during the development of IBL prompted examination of their features as OCPs and their roles in IBL.

### Ly6C^hi^CD11b^hi^ and Ly6C^hi^CD11b^lo^ cells give rise to OCs in vitro and in vivo

**Fig. 2:**
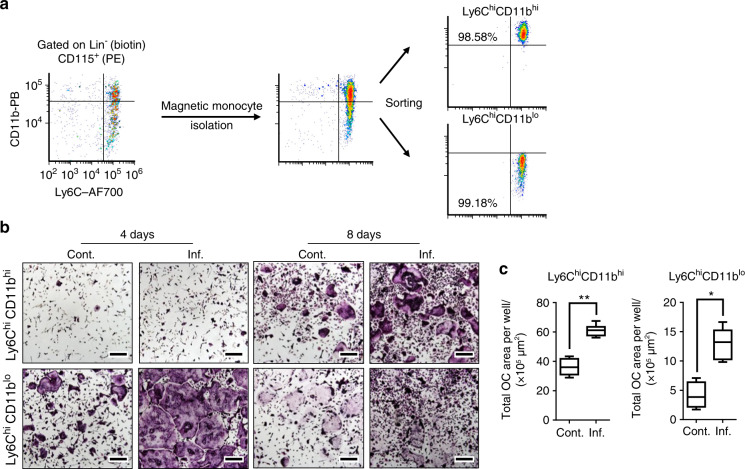
Ly6ChiCD11bhi and Ly6ChiCD11blo cells give rise to OCs in vitro. **a** The isolation and sorting strategy for Ly6C^hi^CD11b^hi^ and Ly6C^hi^CD11b^lo^ cells is presented, and the purity was ≥98% in all samples. **b** Sorted Ly6C^hi^CD11b^hi^ or Ly6C^hi^CD11b^lo^ cells (10^4^) from the BM of control and inflamed mice were cultured with osteoclast differentiation medium for 4 and 8 days. Multinucleated TRAP-stained OCs appeared in Ly6C^hi^CD11b^lo^ cultures after 4 days and in Ly6C^hi^CD11b^hi^ cultures after 8 days (bar: 200 µm). **c** Area quantitation of OCs derived from Ly6C^hi^CD11b^lo^ cells after 4 days and from Ly6C^hi^CD11b^hi^ cells after 8 days in culture. **b**, **c** Control *n* = 5, inflamed *n* = 5, for each cell population; representative results for 5 independent experiments (Mann–Whitney test). Line: median, box: 25th–75th percentile, whiskers: range. *P < 0.05, **P < 0.01

We next tested the osteoclastogenic capabilities of Ly6C^hi^CD11b^hi^ and Ly6C^hi^CD11b^lo^ cells. To this end, these subpopulations were sorted (purity ≥ 98%) from the BM of control or inflamed mice (Fig. 2a). Sorted cells were cultured in OC differentiation medium (RANKL and M-CSF) and then monitored for OCs by tartrate-resistant acid phosphatase (TRAP) staining after 4 and 8 days. Large multinucleated TRAP^+^ OCs were found in the Ly6C^hi^CD11b^lo^ cultures after 4 days, while in the Ly6C^hi^CD11b^hi^ cultures, the TRAP^+^ cells were small and mononucleated (Fig. 2b, left panel). Surprisingly, after 8 days in culture, the Ly6C^hi^CD11b^hi^ cells also gave rise to large multinucleated OCs, showing their delayed osteoclastogenic capability (Fig. 2b, right panel). At this time point, most of the OCs in the Ly6C^hi^CD11b^lo^ cultures had disappeared. OCs derived from either the Ly6C^hi^CD11b^hi^ or Ly6C^hi^CD11b^lo^ population covered a larger area when isolated from the BM of inflamed mice compared to that of noninflamed controls (Fig. 2c). Thus, both the Ly6C^hi^CD11b^hi^ and Ly6C^hi^CD11b^lo^ populations exhibited osteoclastogenic potential, but Ly6C^hi^CD11b^lo^ cells formed OCs more readily than Ly6C^hi^CD11b^hi^ cells in vitro.

**Fig. 3:**
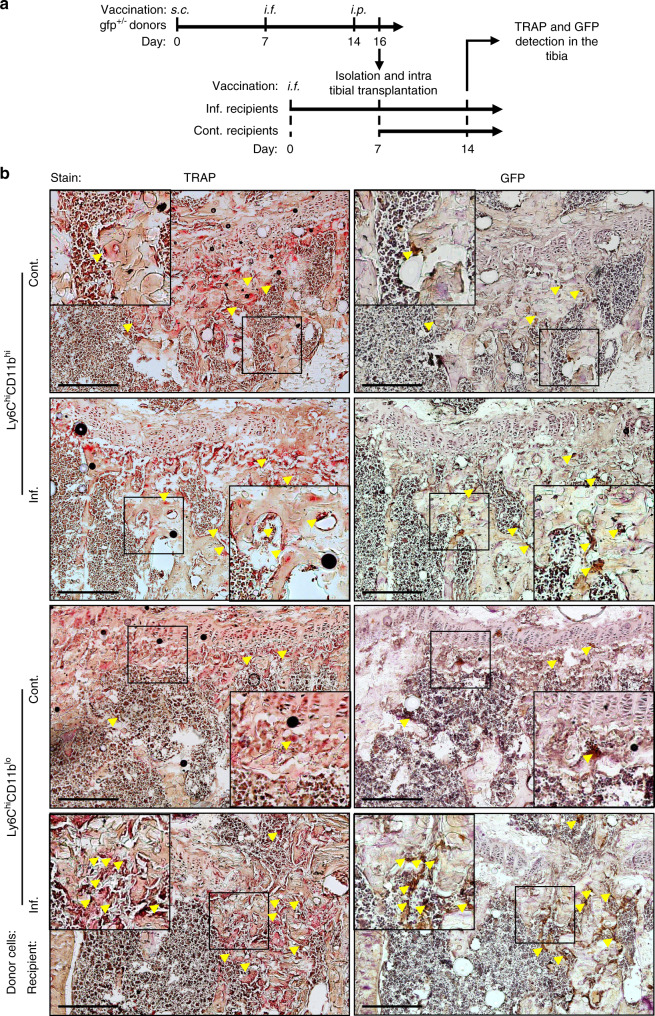
Ly6ChiCD11bhi and Ly6ChiCD11blo cells give rise to OCs in vivo. **a** Sorted Ly6C^hi^CD11b^hi^ or Ly6C^hi^CD11b^lo^ cells (10^6^) from the BM of inflamed *gfp* donor mice were injected into the right tibia of inf. WT recipient mice 7 days after a single *i.f*. vaccination (left foot pad) or into that of sex/age-matched cont. mice. Recipient mice were sacrificed 7 days after transplantation. **b** TRAP staining (left panel) and GFP immunohistochemistry (right panel) of 7-µm consecutive sections from the right tibia of the recipient mice. Black frames are digitally enlarged, and locations positive for both TRAP and GFP are marked by yellow arrows. Scale bar, 400 µm

To determine whether Ly6C^hi^CD11b^hi^ and Ly6C^hi^CD11b^lo^ cells can differentiate into OCs in vivo, we isolated these cells from inflamed *gfp* donors and implanted them into the right tibia of inflamed WT recipients 7 days after a single vaccination administered via the left footpad and into that of noninflamed controls. The recipient mice were sacrificed 7 days after implantation (Fig. 3a). GFP and TRAP were stained in consecutive cryosections of right leg tibiae to identify donor-derived OCs. A small number of GFP^+^ TRAP^+^ cells positioned adjacent to bone tissue were evident in the tibiae from both control and inflamed Ly6C^hi^CD11b^hi^ recipients (Fig. 3b, top panel). Recipients of inflamed Ly6C^hi^CD11b^lo^ cells displayed higher numbers of donor-derived OCs than their respective control recipients (Fig. 3b, bottom panel). Together, these results suggest that both populations are bona fide OCPs, as indicated by the in vitro and in vivo tests. Interestingly, donor-derived OCs were found in the femur adjacent to the implantation site only in control and inflamed recipients of Ly6C^hi^CD11b^hi^ cells (Fig. S3). These results demonstrate that Ly6C^hi^CD11b^hi^ OCPs possess stronger migratory capabilities than Ly6C^hi^CD11b^lo^ OCPs.

We next screened the two subpopulations for the expression of known OCP surface markers. Fractions of both Ly6C^hi^CD11b^hi^ and Ly6C^hi^CD11b^lo^ BM populations were Sca1^lo^ in inflamed mice, but all cells were negative in controls (Fig. S4a, left). Both populations were negative for FLT3 (Fig. S4a, left and middle), and most were CD117^-^ (Fig. S4a, right). The small CD117^+^ fraction was depleted upon monocyte isolation (Fig. S4b), which was performed prior to sorting in all the following ex vivo experiments. Together, these markers exclude both populations from early multipotent progenitors (Lin^-^Sca1^+^CD117^+^), common DC progenitor populations (FLT3^+^)^23^ and cMoPs (Ly6C^hi^CD11b^lo^CD117^+^)^18^. Both populations were CD11c^-^ (Fig. S4c, left), distinguishing them from BM DC populations, which reportedly differentiate into OCs^24,25^. Both populations exhibited low F4/80 expression compared to macrophages, but this expression was higher in Ly6C^hi^CD11b^hi^ cells than in Ly6C^hi^CD11b^lo^ cells (Fig. S4c, right). CX_3_CR1 was strongly expressed in both populations in control mice and downregulated in inflamed mice (Fig. S4d), showing it to be insufficient as a marker for Ly6C^hi^CD11b^hi^ or Ly6C^hi^CD11b^lo^ cells during inflammation, as was previously proposed^16,26^. Both populations expressed CD115 (M-CSF receptor) (Fig. S4d) and TNF-α receptors 1 and 2 (CD120a and CD120b, respectively) (Fig. S4d). Interestingly, both populations were RANK^-^ (Fig. S4d) and thus differed from the committed quiescent OCPs described earlier^27^. However, Ly6C^hi^CD11b^hi^ and Ly6C^hi^CD11b^lo^ cells that were adoptively transferred (*i.v*. injection) from inflamed *gfp* donors were found at low frequencies in the BM of inflamed recipients after 11 days (Fig. S4e) and were RANK^+^ (Fig. S4f). Ly6C^hi^CD11b^lo^ cells shared common markers with previously reported OCPs (Lin^−^Ly6C^hi^CD11b^lo/−^)^16^. Ly6C^hi^CD11b^hi^ cells shared common markers with only heterogeneous populations, such as peripheral monocytes and MDSCs^5,6,28^.

### Immune-inflammatory proteomic profile is observed for Ly6C^hi^CD11b^hi^ cells but not for Ly6C^hi^CD11b^lo^ cells

**Fig. 4:**
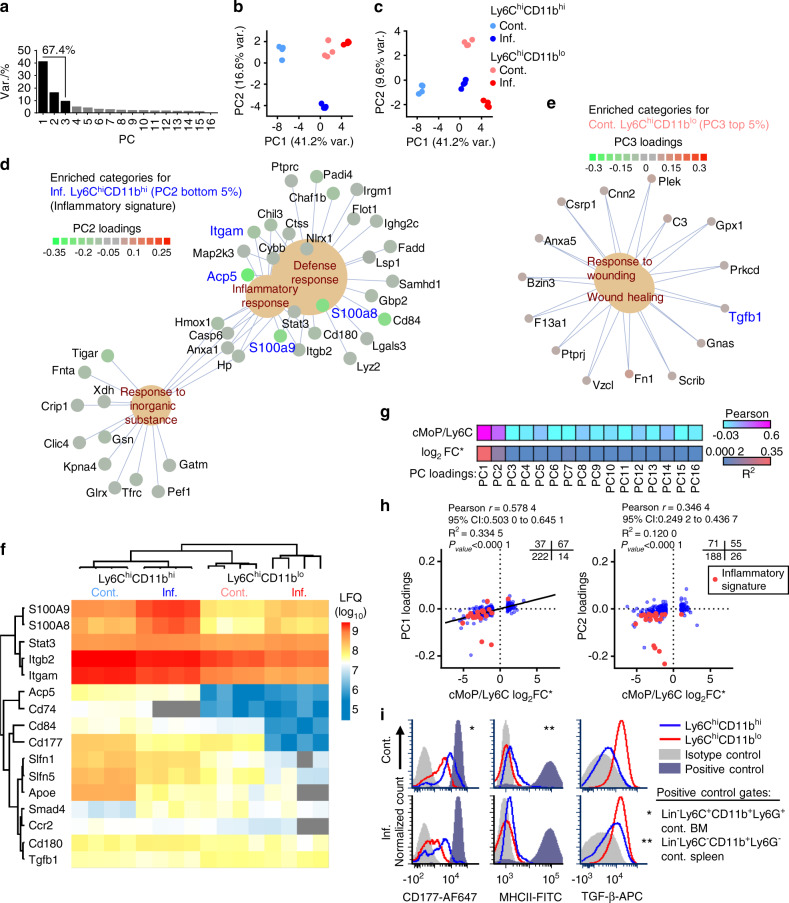
Immune-inflammatory proteomic profile is observed in Ly6ChiCD11bhi cells but not Ly6ChiCD11blo cells. **a** A scree plot for the PCA is presented. **b** Projection plot for PC1 and PC2. **c** Projection plot for PC1 and PC3. **d** Gene concept network of proteins in enriched categories in the bottom 5% of PC2 loading scores (characteristic for inflamed Ly6C^hi^CD11b^hi^ cells). **e** Proteins in enriched categories in the top 5% of PC3 loading scores (characteristic for control Ly6C^hi^CD11b^lo^ cells). The Benjamini–Hochberg FDR was set to 0.05 for all GO analyses (see detailed results in Fig. S4). **f** Heatmap of selected key proteins residing in either the PC1 or PC2 bottom 5% or PC3 top 5% of loading scores. **g** Pearson coefficient and R^2^ heatmap of PC loadings *vs*. the cMoP/Ly6C signature (log_2_FC) reported by Hettinger et al., 2013^18^. **h** Scatterplot of PC1 and PC2 loadings *vs*. the cMoP/Ly6C signature (log_2_FC) (340 proteins detected in both datasets). Quadrant counts indicated (top right). **i** Cell surface expression of the markers CD177, MHCII and TGF-β in control and inflamed Ly6C^hi^CD11b^hi^ and Ly6C^hi^CD11b^lo^ cells. Positive controls are indicated by asterisks

To obtain a better understanding of the molecular programs of Ly6C^hi^CD11b^hi^ *vs*. Ly6C^hi^CD11b^lo^ cells that may explain their functional difference, we used an in-depth quantitative proteomic profiling approach. Ly6C^hi^CD11b^hi^ and Ly6C^hi^CD11b^lo^ cells isolated from the BM of control and inflamed mice (4 biological replicates each) were analyzed and compared to identify differentially expressed proteins. Principal component analysis was performed on the entire dataset (*n* = 6 446 proteins). Principal component 1 (PC1) explained 41.2% of the observed variance, PC2 explained 16.6%, and PC3 explained 9.6% (Fig. 4a). Together, PC1–3 explained 67.4% of the observed variance, indicating substantial differences in the proteome between the two cell populations. PC1 and PC2 segregated control or inflamed Ly6C^hi^CD11b^hi^ cells, respectively, from all other samples (Fig. 4b). PC3 segregated control Ly6C^hi^CD11b^lo^ cells from the rest of the samples (Fig. 4c). Together, these results demonstrate smaller changes in the Ly6C^hi^CD11b^lo^ cell proteomic profile than in the Ly6C^hi^CD11b^hi^ cell proteomic profile during chronic inflammation.

To gain insight into the functional differences among the groups, for each PC, all proteins were ordered according to the respective loadings, and the top or bottom 5% of the proteins were separately subjected to gene ontology (GO) enrichment analysis (see the Methods section). Proteins with low PC1 loadings (associated with control Ly6C^hi^CD11b^hi^ cells) were enriched for immune cell-related terms such as locomotion, response to external stimulus and phagocytosis (Fig. S5a). The top scoring PC1 proteins (associated with all samples except control Ly6C^hi^CD11b^hi^ cells) were enriched for ribosomal and DNA replication terms (Fig. S5b). For PC2, enrichment was found only among the bottom 5% of the proteins (inflamed Ly6C^hi^CD11b^hi^ cells) for inflammation-related terms, such as defense response, inflammatory response, and response to inorganic substances (Fig. 4d). The proteins in these categories made up a unique inflammatory signature in inflamed Ly6C^hi^CD11b^hi^ cells, with key proteins such as ITGAM (CD11b), the OC-related phosphatase ACP5 (TRAP) and the proinflammatory S100A8/A9 proteins. The top scoring PC3 proteins (control Ly6C^hi^CD11b^lo^ cells) were enriched for wound healing-related terms, with key proteins such as transforming growth factor β (TGF-β), fibronectin 1 (FN1) and complement C3 (Fig. 4e). According to the enriched functional annotations, we termed PC1 the maturation component and PC2 the inflammatory component, and these were considered to be the two components that separated the Ly6C^hi^CD11b^hi^ subpopulation from the Ly6C^hi^CD11b^lo^ subpopulation (Fig. S5 includes the full results for the GO analysis). The S100A8/A9 proteins were more prominently expressed in Ly6C^hi^CD11b^hi^ cells than in Ly6C^hi^CD11b^lo^ cells and were upregulated in Ly6C^hi^CD11b^hi^ cells during chronic inflammation (Fig. 4f). Other noteworthy proteins specific for Ly6C^hi^CD11b^hi^ cells included STAT3, ITGB2 (CD18), ITGAM (CD11b), ACP5, CD74 (major histocompatibility complex II (MHCII)), the adhesion proteins CD84 and CD177, the Schlafen family members SLFN1 and SLFN5, apolipoprotein E (APOE), the chemokine receptor CCR2 and the toll-like receptor-associated protein CD180. TGFB1 (TGF-β) and the downstream protein SMAD4 were preferentially expressed by Ly6C^hi^CD11b^lo^ cells (Fig. 4f). These GO analyses identified an immune-inflammatory proteome in Ly6C^hi^CD11b^hi^ cells and a homeostatic profile in Ly6C^hi^CD11b^lo^ cells.

To understand the positions of both subsets on the differentiation scale of Ly6C^hi^ monocytes, we compared our proteomic data to a previously published dataset that compared the immature cMoP population to the generally mature Ly6C^hi^ monocytic population (cMoP/Ly6C signature)^18^. We found 340 proteins common to both datasets. The maturation component (PC1) and the inflammatory component (PC2) loadings best correlated with the log_2_FC of the cMoP/Ly6C signature, and no correlation was found with any of the other PCs (Fig. 4g). The maturation component loadings positively correlated with the cMoP/Ly6C signature (Fig. 4h, left graph), with a set of 222 Ly6C-specific proteins with negative loadings (control Ly6C^hi^CD11b^hi^ cells) and 67 cMoP-specific proteins with positive loadings. This result shows that control Ly6C^hi^CD11b^hi^ cells resemble mature Ly6C monocytes, while inflamed Ly6C^hi^CD11b^hi^ and Ly6C^hi^CD11b^lo^ cells derived from both control and inflamed mice are more similar to immature cMoPs. The inflammatory component loadings positively correlated with the cMoP/Ly6C signature (Fig. 4h, right graph), with a set of 188 Ly6C-specific proteins with negative loadings (inflamed Ly6C^hi^CD11b^hi^). Most proteins belonging to the inflammatory signature were Ly6C specific (Fig. 4h), further demonstrating the more mature immune-inflammatory properties of Ly6C^hi^CD11b^hi^ cells.

We validated the expression of the surface markers CD177, MHCII and TGF-β derived from the proteomic analysis (Fig. 4i). CD177 was higher in Ly6C^hi^CD11b^hi^ cells than in Ly6C^hi^CD11b^lo^ cells under control and inflammatory conditions; however, both populations expressed this marker. Ly6C^hi^CD11b^hi^ cells expressed low levels of MHCII, but this expression was higher than that in Ly6C^hi^CD11b^lo^ cells. We found high and stable expression of cell-surface TGF-β in Ly6C^hi^CD11b^lo^ cells, lower levels in inflamed Ly6C^hi^CD11b^hi^ cells and none in control Ly6C^hi^CD11b^hi^ cells. The relative expression levels of CD177 and MHCII in Ly6C^hi^CD11b^hi^ cells and those of TGF-β in Ly6C^hi^CD11b^lo^ cells under normal and inflammatory conditions (Fig. 4i) were in accordance with the results in the presented heatmap of selected key proteins (Fig. 4f).

In conclusion, Ly6C^hi^CD11b^lo^ cells exhibited only a small change in their proteomic profile during chronic inflammation and henceforth are termed homeostatic OCPs (hOCPs). Ly6C^hi^CD11b^hi^ cells, on the other hand, exhibited a profound change during chronic inflammation, which reprogrammed them to be less mature, similar to hOCPs and cMoPs, and imparted a unique inflammatory signature. Therefore, these cells are herein termed iOCPs.

### iOCPs display an immature functional phenotype during chronic inflammation

**Fig. 5:**
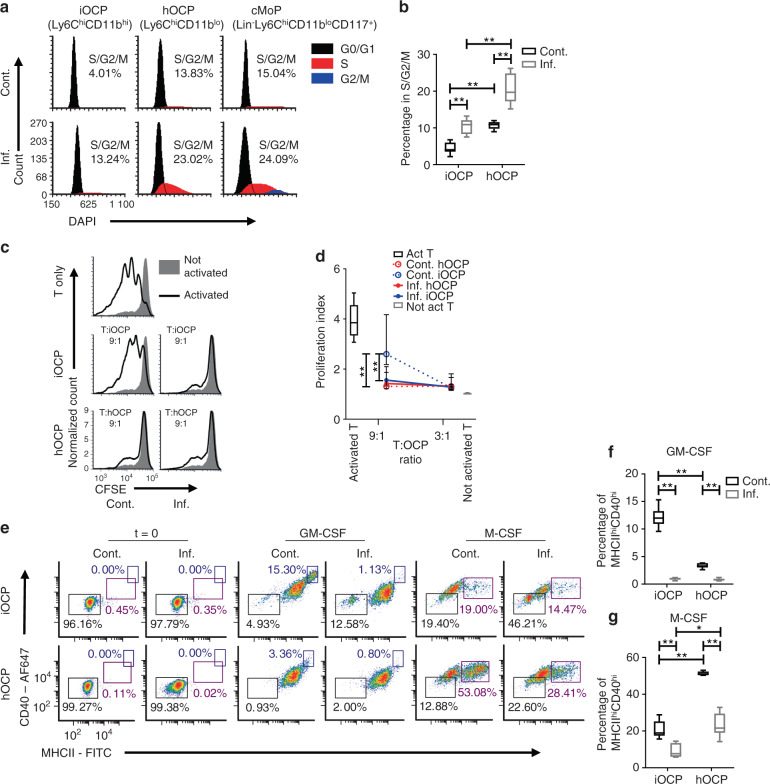
iOCPs and hOCPs are functionally distinct myeloid precursor populations. **a** Cell cycle staining of iOCPs (Ly6C^hi^CD11b^hi^) and hOCPs (Ly6C^hi^CD11b^lo^) in the BM of control and inflamed mice. Lin^-^Ly6C^hi^CD11b^lo^CD117^+^ cMoPs were used as a positive control, and iOCPs and hOCPs were identified from the CD117^-^ gate. **b** The frequency of cells in the S/G2/M phase. *n* = 6 for each group, representative of two independent experiments. **c** Representative plots for the T-cell proliferation suppression assay at a 9:1 T cell:OCP ratio with iOCPs and hOCPs. **d** The proliferation indices at 3:1 and 9:1 T cell:OCP ratios are presented. *n* = 5 for each group, representative results for 2 independent experiments. **e** MHCII and CD40 expression of iOCPs and hOCPs sorted from the BM of control or inflamed mice after 3 days in culture with GM-CSF or M-CSF. **f** The fraction of MHCII^hi^CD40^hi^ differentiated cells in GM-CSF-containing cultures. **g** The frequency of MHCII^hi^CD40^hi^ differentiated cells in M-CSF-containing cultures. *n* = 6 for each group, representative results for 3 independent experiments. Line/circle: median, box: 25th-75th percentile, whiskers: range. *P < 0.05, **P < 0.01 (Mann–Whitney test and Holm multiplicity correction)

We proceeded to examine functional differences related to monocytic maturity to verify our findings from the proteome analysis. The fraction of cycling cells (in the S/G2/M phase) was higher in hOCPs than in iOCPs in general, with comparable values between hOCPs and Lin^−^Ly6C^hi^CD11b^lo^CD117^+^ cMoPs (Fig. 5a). The higher cell cycle activity of hOCPs demonstrates their similarity to myeloblasts and progenitors such as cMoPs. Both populations exhibited a proportional 2-fold increase in cycling cells during chronic inflammation (Fig. 5b). This shows that iOCPs gain proliferative activity during chronic inflammation, in accordance with their maturation state described in the proteome analysis.

The ability to suppress T-cell proliferation is characteristic of immature monocytes^29,30^. We tested that function in iOCPs and hOCPs as a reflection of their maturity. Only control iOCPs did not suppress T-cell proliferation in cocultures with a 9:1 T cell:OCP ratio but gained suppressive activity in inflamed mice, while control and inflamed hOCPs were suppressive at this ratio (Fig. 5c). However, both hOCPs and iOCPs suppressed T-cell proliferation at a 3:1 T cell:OCP ratio, whether isolated from the BM of control or inflamed mice, showing that both populations are inherently suppressive (Fig. 5d). We conclude that hOCPs are more suppressive than iOCPs under normal conditions. However, during chronic inflammation, iOCPs gain suppressive activity and become as suppressive as hOCPs, further demonstrating the similarity induced by chronic inflammation.

We next tested the ability of iOCPs and hOCPs to increase maturation marker expression in response to GM-CSF or M-CSF. iOCPs and hOCPs from the BM of control or inflamed mice were cultured with GM-CSF or M-CSF for 3 days. The expression of MHCII and CD40 was monitored, and MHCII^hi^CD40^+^ cells were considered to be differentiating cells (Fig. 5e). GM-CSF-induced differentiation was maximal in control iOCPs and minimal in control hOCPs, while this differentiation was completely blocked in inflamed iOCPs and hOCPs (Fig. 5f). M-CSF-induced differentiation was observed in all cultures, but mostly in control hOCP cultures; this differentiation was also blocked in inflamed iOCPs and hOCPs (Fig. 5g). Interestingly, iOCPs were biased toward response to GM-CSF and hOCPs toward response to M-CSF. These differential responses to the myeloid cell growth factors GM-CSF and M-CSF demonstrate the biased cell fates of iOCPs and hOCPs, further showing that they are distinct populations. Overall, hOCPs display an immature phenotype under control and inflammatory conditions, while iOCPs are reprogrammed toward an immature phenotype during chronic inflammation, as described in the proteome analysis.

### iOCP-derived OCs are more active and express more resorption-related proteins than hOCP-derived OCs

**Fig. 6:**
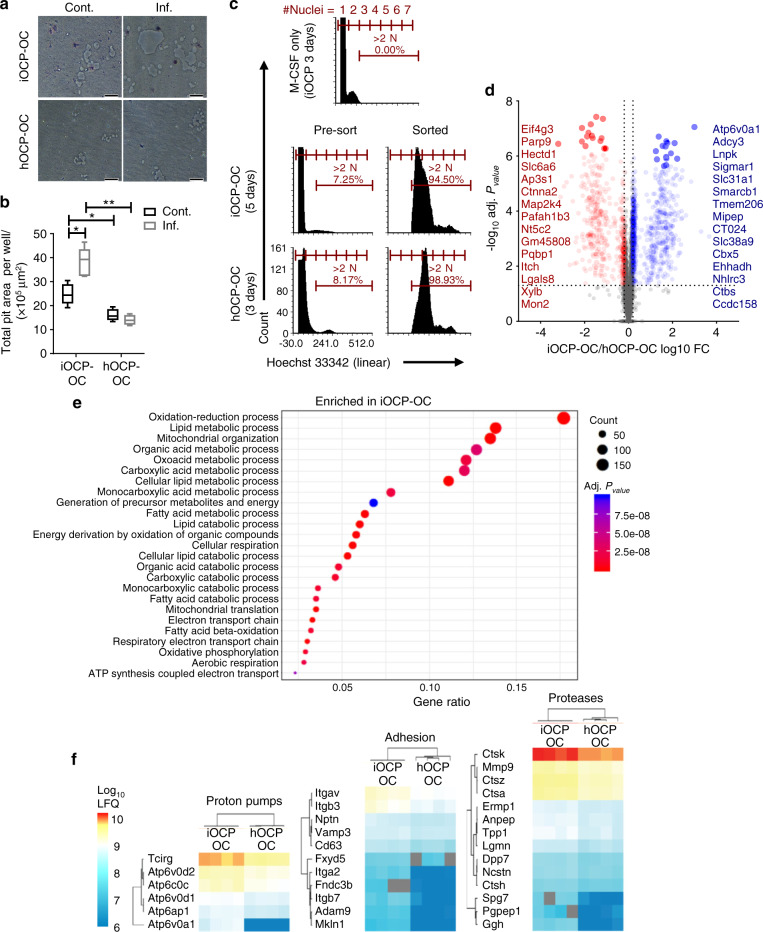
iOCP-derived OCs are more active and express more resorption-related proteins than hOCP-derived OCs. **a** Sorted iOCPs or hOCPs (5 × 10^4^) from the BM of control and inflamed mice were cultured on Osteo assay surface plates in OC differentiation medium to generate OCs (iOCP-OCs and hOCP-OCs, respectively). The cells were cultured for 10 days until OC exhaustion (bar: 50 µm). **b** Total pit area per well represents the total resorption potential of a fixed number of precursors. *n* = 5 for each group, representative results for three independent experiments. Line: median, box: 25th–75th percentile, whiskers: range. *P < 0.05, **P < 0.01 (Mann–Whitney test and Holm multiplicity correction). **c** hOCPs and iOCPs sorted from the BM of inflamed mice were cultured for 3 and 5 days, respectively, with RANKL and recombinant M-CSF to generate iOCP-OCs and hOCP-OCs. The cultures were harvested, and mature OCs were sorted as cells with more than two nuclei identified by Hoechst 33342 staining. The threshold was set according to iOCPs cultured with only M-CSF for 3 days. **d** Top 15 differentially expressed proteins between iOCP-OCs and hOCP-OCs. **e** Enriched gene ontology biological process terms among significant iOCP-OC proteins. hOCP-OCs were enriched in only DNA replication (GO: 0006260). The Benjamini–Hochberg FDR was set to 0.05 for the GO analysis. **f** Expression heatmaps of OC activity-related proton pumps, adhesion molecules and proteases, which were significantly more highly expressed in iOCP-OCs. Four biological replicates were used (Limma, FDR = 0.05)

As we identified iOCPs but not hOCPs as inflammation-responsive cells, we next tested the function of iOCPs as OCPs under control and inflammatory conditions. iOCPs or hOCPs isolated from the BM of control or inflamed mice were cultured in OC differentiation medium on Osteo assay surface plates for 10 days to generate OCs, iOCP-OCs and hOCP-OCs, respectively. We used a larger number of cells in these cultures (5 × 10^4^) to hasten OC formation, and thus, after 10 days, all OCs in the cultures were exhausted. The total resorptive activity exhibited by OCs derived from a fixed number of OCPs until OC exhaustion in culture reflects the number, longevity and activity of the OCs and therefore represents the total resorption potential. Although resorption pits were visible in all cultures (Fig. 6a), larger pit areas were measured in iOCP-OC cultures from both the control and inflamed groups compared to hOCP-OC cultures (Fig. 6b). Moreover, the largest pit areas were observed in the inflamed iOCP-OC cultures, showing that iOCP-OCs gained resorption potential during chronic inflammation. The hOCP-OCs displayed low resorption potential, which was unchanged during chronic inflammation, showing that similar to iOCPs, only iOCP-OCs change cellular behavior during chronic inflammation.

We next investigated the molecular differences between iOCP-OCs and hOCP-OCs. To this end, hOCPs and iOCPs isolated from the BM of inflamed mice were cultured in OC differentiation medium for 3 and 5 days, respectively. To overcome culture asynchronicity and to generate pure OC samples, mature OCs were sorted from the cultures by Hoechst 33342 nuclear staining, as previously described^31^. Mature OCs were detected as cells containing three or more nuclei, and iOCPs cultured with only M-CSF for 3 days were used as a control to set the threshold above two nuclei (Fig. 6c). The fraction of mature OCs, containing 3–6 nuclei, ranged between 7% and 12% in all samples. We used comparative proteomic profiling to elucidate the differences between inflamed iOCP-OCs and hOCP-OCs (4 biological replicates each). A total of 5 530 proteins were detected, of which 692 were preferentially expressed by iOCP-OCs and 1 083 by hOCP-OCs (Limma, Benjamini–Hochberg false discovery rate (FDR) = 0.05) (Fig. 6d). Significant iOCP-OC-specific proteins were enriched in GO categories including mitochondrial, lipid and acid metabolic processes (Fig. 6e), while hOCP-OC-specific proteins were enriched in only DNA replication (GO:0006260). This shows that iOCP-OCs are more metabolically active than hOCP-OCs during chronic inflammation. We then searched the data for proton pumps, adhesion molecules and proteases, which are protein classes that have been previously associated with OC activity. The subunits and associated proteins of the V-type proton pump complex were all more highly expressed in iOCP-OCs, with the OC-specific TCIRG and ATP6v0d2 subunits most prominently expressed (Fig. 6f, left panel). Most adhesion molecules were preferentially expressed by iOCP-OCs, with components of the OC-specific αVβ3 integrin complex (ITGAV and ITGB3) exhibiting the highest expression (Fig. 6f, middle panel). Protease expression was also higher in iOCP-OCs, with OC-specific CTSK exhibiting the highest expression (Fig. 6f, right panel). Together, these data show that iOCP-OCs are more active than hOCP-OCs, in both metabolism and bone resorption.

### S100A8/A9 proteins augment differentiation of iOCPs but not that of hOCPs toward highly active osteoclasts

**Fig. 7:**
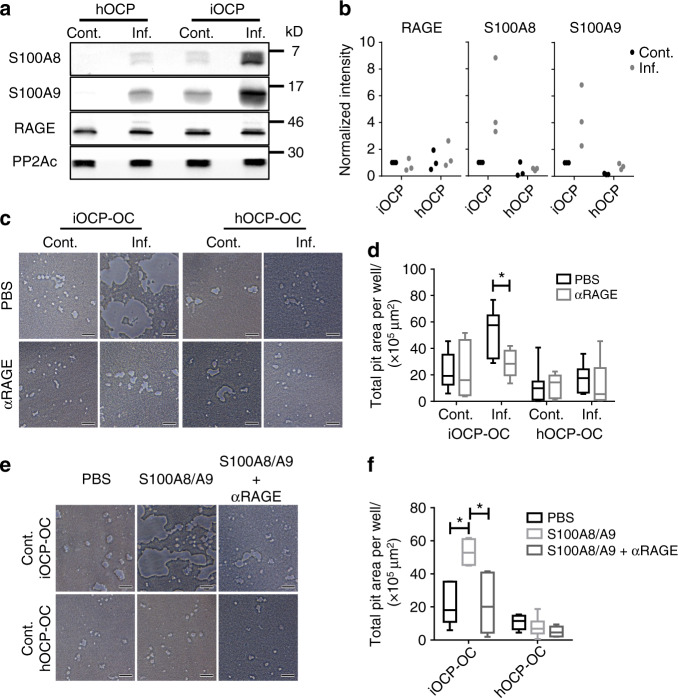
S100A8/A9 proteins augment differentiation and osteoclastic activity derived from iOCPs but not from hOCPs. **a** Expression of S100A8, S100A9 and RAGE in sorted control and inflamed BM iOCPs and hOCPs. The protein phosphatase 2A catalytic subunit (PP2Ac) is shown as a loading control. **b** Densitometry measurements of three biological repeats (no statistical analysis presented). **c** Sorted control and inflamed BM iOCPs and hOCPs (5 × 10^4^) were cultured on the Osteo assay surface with or without anti-RAGE blocking antibodies (bar: 50 µm). **d** Pit area quantitation. **e** Sorted iOCPs and hOCPs (5 × 10^4^) from the BM of control mice were cultured on the Osteo assay surface in combination with a recombinant S100A8/A9 heterodimer and anti-RAGE blocking antibodies as indicated (bar: 50 µm). **f** Pit area quantitation. **c**–**f** Depict representative results for two independent experiments, *n* = 5 for each group. Line: median, box: 25th-75th percentile, whiskers: range. *P < 0.05 (Mann–Whitney test and Holm multiplicity correction)

To elucidate the mechanism underlying the response of iOCPs to inflammatory signals that lead to the differentiation of OCs with high resorptive activity, we focused on key proteins belonging to the inflammatory signature obtained when analyzing inflamed iOCPs. The S100A8/A9 proteins were chosen due to their significant contribution to the inflammatory component (PC2) (Fig. 4d), their abundant expression in inflamed iOCPs (Fig. 4f), and their known role in the regulation of inflammatory processes and the immune response^20^. Analyzing the expression levels of these proteins in the different OCP subpopulations revealed that only iOCPs substantially increased S100A8/A9 expression during chronic inflammation, while control and inflamed hOCPs exhibited low expression of these proteins (Fig. 7a, b). The receptor for S100A8/A9, RAGE, was found to be equally expressed by both OCP populations in inflamed and control mice. Protein phosphatase 2A catalytic subunit (PP2Ac) was used as a loading control.

We further tested whether the RAGE-S100A8/A9 axis affects the differentiation of iOCPs and hOCPs into OCs. We used combinatorial blockade of S100A8, S100A9 and RAGE in OC cultures of iOCPs and hOCPs isolated from inflamed BM to determine the involvement of each protein in osteoclastogenesis. While blocking S100A8 or S100A9 separately did not affect the osteoclastogenesis of iOCPs or hOCPs, simultaneous blockade of S100A8 and S100A9 and/or RAGE inhibited the OC formation of only iOCPs, not that of hOCPs (Fig. S6a, b). Blocking RAGE abrogated the enhanced resorption observed in inflamed iOCP cultures but did not affect that in control iOCP cultures or cultures of hOCPs from inflamed or control mice (Fig. 7c, d). Since iOCPs but not hOCPs express endogenous S100A8/A9, we further tested whether control iOCPs and hOCPs can respond to exogenously added S100A8/A9 with enhanced OC activity. Control iOCPs generated iOCP-OCs with enhanced activity in response to S100A8/A9, and this effect was abrogated by RAGE blockade, while control hOCPs did not respond to S100A8/A9 or RAGE blockade (Fig. 7e, f). Taken together, these results show that only iOCPs respond to autocrine and exogenous S100A8/A9 via RAGE by differentiating into highly resorptive OCs and that this interaction does not affect hOCPs.

### Reduced accumulation of iOCPs but not hOCPs in inflamed *tnf-α*^*−/−*^ mice correlates with attenuated IBL

**Fig. 8:**
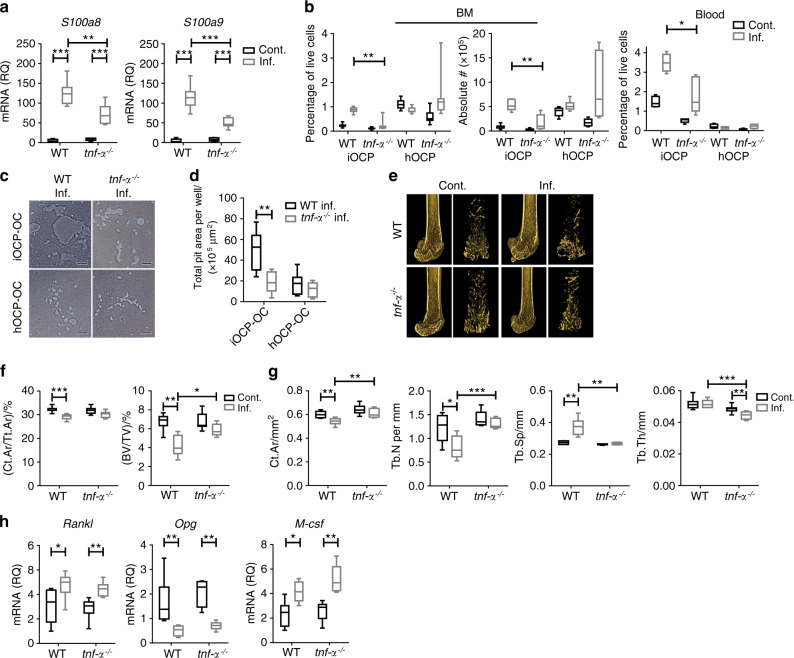
TNF-α ablation abrogates IBL by targeting iOCPs but not the hOCP response during chronic inflammation. **a** Expression of *S100a8* and *S100a9* transcripts in the right tibia of control and inflamed WT and *tnf*-*α*^*−/−*^mice. **b** iOCP and hOCP frequencies and absolute numbers in the BM of control and inflamed WT and *tnf*-*α*^*−/−*^ mice (left panel) and the fractions in the peripheral blood (right panel). **c**. Sorted iOCPs and hOCPs (5 × 10^4^)- from the BM of inflamed WT and *tnf*-*α*^*−/−*^ mice cultured on the Osteo assay surface (bar: 50 µm). **d** Quantitation of total pit area/well. **e** Representative 3D modeling from femoral microCT scans of control and inflamed WT and *tnf*^*-*^*-α*^*−/−*^ mice. **f** Cortical cross-sectional area fraction (Ct.Ar/Tt.Ar) and trabecular BV/TV. **g** Morphometric indices; Ct.Ar, Tb.N, Tb.Sp and Tb.Th. **h** Expression of *Rankl*, *Opg* and *M-csf* in right tibiae. **a**, **b** and **e**–**h** WT: control *n* = 7, inflamed *n* = 7; *tnf*-*α*^*−/−*^: control *n* = 8, inflamed *n* = 8; representative results for three independent experiments (Mann–Whitney test and Bonferroni multiplicity correction for **a** and **f**–**h**, Holm multiplicity correction for **b**. **c**, **d:** *n* = 8 for each group, representative results for three independent experiments (Mann–Whitney test and Holm multiplicity correction). Line: median, box: 25th–75th percentile, whiskers: range. *P < 0.05, **P < 0.01, ***P < 0.001, ****P < 0.000 1

We have previously shown that during chronic inflammation, TNF-α is the master regulator of myelopoiesis and functions by controlling the S100A8/9-RAGE axis in immature myeloid cells^20^. Therefore, we next assessed the responses of iOCPs and hOCPs during chronic inflammation in the absence of TNF-α. To this end, we compared the responses of the two OCP subpopulations during chronic inflammation in WT and *tnf-α*^*−/−*^ mice. We found reduced levels of S100A8/A9 transcripts in the tibiae of inflamed *tnf-α*^*−/−*^ mice compared to inflamed WT mice (Fig. 8a), showing less favorable conditions for iOCP-OC generation in the *tnf-α*^*−/−*^ mice and suggesting that S100A8/A9 are partially regulated by TNF-α. Inflamed *tnf-α*^*−/−*^ mice showed a reduced percentage and absolute number of BM iOCPs, while the number and percentage of hOCPs were unchanged (Fig. 8b, left panel). Fewer iOCPs circulated in the blood of inflamed *tnf-α*^*−/−*^ mice than in that of inflamed WT mice, while hOCPs were not found in the blood (Fig. 8b, right panel). We then assessed the activity of iOCP-OCs and hOCP-OCs generated from the BM of inflamed *tnf-α*^*−/−*^ and WT mice. iOCP-OCs from inflamed *tnf-α*^*−/−*^ mice displayed lower resorptive activity than the respective WT iOCP-OCs, while for hOCP-OCs, the resorptive activity was low and unchanged between *tnf-α*^*−/−*^ and WT mice (Fig. 8c, d). These results show that the response of iOCPs but not that of hOCPs during chronic inflammation was inhibited in the absence of TNF-α.

IBL was evident in chronically inflamed WT mice but not in *tnf-α*^*−/−*^ mice (Fig. 8e). The cortical bone fraction (Ct.Ar/Tt.Ar) and trabecular bone volume fraction (BV/TV) were lower in inflamed WT mice but not in *tnf-α*^*−/−*^ mice (Fig. 8f). The microarchitecture was significantly altered by chronic inflammation in WT mice but not in *tnf-α*^*−/−*^ mice; the Ct.Ar and Tb.N were significantly decreased, and Tb.Sp was significantly increased in inflamed WT mice but not in inflamed *tnf-α*^*−/−*^ mice (Fig. 8g). Tb.Th was unchanged in WT mice but was decreased in inflamed *tnf-α*^*−/−*^ mice (Fig. 8g, rightmost panel). Overall, WT mice suffered severe trabecular and cortical bone loss, while *tnf-α*^*−/−*^ mice developed mild IBL, displaying a partial loss of trabecular bone and no cortical bone loss, as was previously described for ovariectomized mice^32^. The response of iOCPs but not that of hOCPs was ablated in the absence of TNF-α, and IBL was correlatively attenuated, suggesting a more central role for iOCPs than for hOCPs in the induction of IBL. Interestingly, the expression levels of *Rankl*, *Opg,* and *M-csf* transcripts in the tibiae of inflamed WT and *tnf-α*^*−/−*^ mice were similar (Fig. 8h), thus not correlating with the attenuation of IBL. These findings further suggest that the change in the OCP populations, not the osteoclastogenic signals, is the major contributor to IBL.

## Discussion

IBL is common to a wide range of diseases characterized by developing chronic inflammation. To understand the link between bone loss and chronic inflammation, the robust changes in myelopoiesis and the distribution of osteoclastogenic BM myeloid populations must be considered. This warrants examination of the specific OCP subsets that respond to the inflammatory settings that ultimately cause IBL. Various general myeloid populations were shown to form active OCs, such as BM CD115^+^CD117^+^ cells and MDSCs, which consist of several cell types. Overlapping these populations are the abundant BM monocytes with a Ly6C^hi^CD11b^lo^ phenotype, which were previously shown to be the most potent OCP population in vitro and in normal and arthritic mice^16,33^. These cells were our first candidates for studying OCPs in chronic inflammation. Unexpectedly, we found these precursors to be unchanged during chronic inflammation. Within the Ly6C^hi^ monocytic compartment, we found that CD11b^hi^ monocytes also formed OCs in vitro and in vivo and constituted a separate bona fide OCP subset. This subset was maintained at low numbers under normal conditions and had low OC formation potential, rendering them unidentifiable in such settings. Nevertheless, this subset expanded dramatically and became a potent OCP population during chronic inflammation, accordingly termed iOCPs (inflammatory). This contrasted with the highly osteoclastogenic CD11b^lo^ subset, which was prevalent in the BM under normal conditions but did not change in number or resorption potential during chronic inflammation, accordingly termed hOCPs (homeostatic).

Although closely related, iOCPs and hOCPs display different proteomic signatures, which mark each subset with its unique attributes. hOCPs are less mature and display monoblast-like behavior, as they are more proliferative and immunosuppressive. iOCPs are more mature and display an immune cell-related proteomic signature consisting of innate immune response-related receptors, downstream mediators, and migration proteins (the maturation component). The migratory capability of iOCPs was further supported by their observed ability to migrate out of the implantation site in the tibia and reach the adjacent femur. This is in concordance with previous reports that peripheral OCPs migrating out of the BM display high expression of CD11b^28^. Under normal conditions, iOCPs are dramatically different from hOCPs and display low osteoclastogenic potential. During chronic inflammation, iOCPs become more similar to hOCPs, both in their proteome and functionality, in terms of their cell cycle activity and their ability to suppress T-cell proliferation. Despite the similarity between the two subsets, our proteomic profiling highlighted that chronic inflammation imprinted a unique inflammatory signature in iOCPs (the inflammatory component), which was absent in hOCPs. This was further corroborated by the specific response of iOCPs but not hOCPs to key inflammatory signals, such as the S100A8/A9 proteins. The iOCP-specific response to S100A8/A9 drives the generation of highly active OCs during chronic inflammation. hOCPs are insensitive to such signals, although they do express the corresponding receptor RAGE. Thus, the inflammatory component uniquely controls the osteoclastogenic cell fate of iOCPs. Furthermore, iOCPs enhance their osteoclastogenic and resorption potentials in hand with the loss of maturity, corroborating the link between immaturity and osteoclastogenicity, as was suggested before^34^.

It was previously shown that CD11b^hi^ monocytes are generated from CD11b^−/lo^ progenitors^18,19^. We could not find evidence to support or disprove that hOCPs give rise to iOCPs or vice versa. However, such differentiation does not affect our main claim that only inflammation-induced iOCPs generate highly active OCs.

The observation that both iOCPs and hOCPs, which together comprise the whole Ly6C^hi^ population, are osteoclastogenic identifies the entire BM Ly6C^hi^ monocytic population as a general OCP population. These monocytes make up the majority of BM OCPs during chronic inflammation and under normal conditions due to their high abundance relative to the abundances of other specific OCP subsets, such as DCs and QOPs. Most classic OCP markers are commonly expressed by iOCPs and hOCPs. We found the markers CD115, CD117, Ly6C, CX_3_CR1 and RANK to be insufficient for discriminating between iOCPs and hOCPs. We therefore proposed, according to our proteomic profiling findings and subsequent validation, that the relative expression of CD177, MHCII and the S100A8/A9 proteins could serve as potential iOCP markers, while that of TGF-β could serve as an hOCP marker. Further studies are required to identify additional iOCP markers for the prediction and/or evaluation of IBL development in the context of human disease.

A surprising result was the discrepancy between the osteoclastogenic and resorption potentials. iOCPs displayed low osteoclastogenic potential both in vitro and in vivo yet had high resorption potential, which was enhanced by chronic inflammation, while hOCPs displayed the opposite features. As the kinetics of osteoclastogenesis depend on the proximity between the precursors to allow fusion, proliferative hOCPs showed an advantage and thus formed OCs more potently. This is in accordance with previous reports showing that Ly6C^hi^CD11b^lo^ OCPs display rapid OC formation in vitro^16,33^ and that CD11b is a negative regulator of osteoclastogenesis^35^. However, the rate of osteoclastogenesis is unrelated to the activity of the resultant OCs, which depends on attributes of the forming OCs, *i.e*., the maximal number of OCs generated, their lifespan and, as we found, the resorptive activity of each OC. This emphasizes the need for a functional assay to determine the resorption potential of OCs arising from different OCP subsets. Furthermore, it highlights the central role of the small and seemingly less potent iOCP subset in modulating bone resorption.

Our observation that *tnf-α*^*−/−*^ mice displayed attenuated IBL during chronic inflammation is consistent with the literature, as these mice were previously shown to be resistant to ovariectomy-induced bone loss^32^. Although the ‘protective’ effect of TNF-α ablation in IBL can be mediated through more than one process, its correlation with the inhibition of only iOCPs, not hOCPs, is suggestive of the relevance of the former and not the latter to IBL in vivo. Moreover, the high osteoclastogenic signals in inflamed *tnf-α*^*−/−*^ mice do not correlate with the attenuation of IBL, but the lower S100A8/A9 signals do, further linking the iOCP response to IBL. Genetic targeting or tracing of the two OCP subsets by utilizing population-specific promotors could verify this correlation and prove the relevance of these subpopulations to IBL in vivo; however, none have been found thus far. This point should be further investigated.

The general BM CD115^+^ monocytic population is multipotent and has been shown to differentiate into macrophages, OCs, DCs or glial cells, depending on cytokine exposure^36,37^. This general population consists of several types of monocytes in different stages of differentiation, which may be lineage restricted. We found that the APC differentiation patterns of iOCPs and hOCPs were biased toward responsiveness to GM-CSF or M-CSF, respectively, suggesting a lineage restriction. Nevertheless, during chronic inflammation, these opposite tendencies in immune effector differentiation were inhibited for both subsets, along with an increase in the osteoclastogenic cell fate for iOCPs only. These findings emphasize the heterogeneity of Ly6C^hi^ monocytes under normal conditions and their plasticity during chronic inflammation.

Our results conflict with a report by Charles et al., which showed that Ly6C^hi^CD11b^lo^ cells are bona fide OCPs that expand in the BM of arthritic SKG mice^16^; however, these cells did not expand in our models. This discrepancy may arise from pathological differences, as arthritic mice display localized bone inflammation, while our systemically inflamed mice do not. Nevertheless, a statistically nonsignificant tendency for hOCP expansion was evident in our data but negligible compared to iOCP accumulation. In contrast to our results, Charles et al. did not find any osteoclastogenesis in Ly6C^hi^CD11b^hi^ cells. However, they isolated OCP subsets from nonarthritic SKG and WT mice, and for that reason were unable to generate OCs from these inflammation-inducible OCPs.

In conclusion, we propose that under normal conditions, most OCs occupying the bone surface are derived from hOCPs, which comprise the dominant population in the normal BM. During chronic inflammation iOCPs are induced, and highly active iOCP-OCs occupy a larger proportion of the bone surface at the expense of less active hOCP-OCs. This may result in increased overall resorptive activity and IBL.

IBL is associated with a myriad of bone disorders, from fracture susceptibility to enhanced BM metastases in cancer patients. Our findings reveal IBL as a general and systemic complication of chronic inflammation, in which alterations in myelopoiesis and OCP populations contribute to the pathological process. Similar to chronic inflammation, IBL is a silent complication that can only be detected post-factum, when bone mass is already lost. Thus, the identification of specific OCPs, which are evident in the blood as well, and the pathways governing their differentiation into OCs offers novel options for biomarkers to predict and monitor IBL development in diseases characterized by chronic inflammation before any disabling bone loss occurs. Our findings uncover new potential therapeutic targets to prevent and treat IBL based on the possibility of specifically targeting iOCPs while leaving hOCPs unharmed. This approach could prove beneficial compared to currently used treatments that all target mature osteoclasts including bisphosphonates, which suppress bone turnover^38^, and anti-RANKL antibodies, which cause a high turnover rebound response^39^.

## Materials and methods

### Mice

C57BL/6J female mice (9 weeks of age) were purchased from Harlan Israel and housed at the Hebrew University specific pathogen-free facility. *tnf-α*^−/−^ C57BL6.129S-*Tnf*^tm1Gkl^/J mice were obtained from The Jackson Laboratory. *gfp* transgenic C57BL/6-Tg(UBC-GFP)30Scha/J mice were a kind gift from Prof. Reuven Or (Hadassah Medical Center, Jerusalem Israel). Animal use followed protocols approved by the Hebrew University-Hadassah Medical School Institutional Animal Care and Use Committee.

### Induction of chronic inflammation by repetitive vaccination

Mice were vaccinated with 50 µg heat-killed *Mycobacterium tuberculosis* H37 Ra (BCG) (Difco Laboratories, Cat#: 231141) once per week over 16 days (Days 0, 7, and 14). The first injection (Day 0) was emulsified in incomplete Freund’s adjuvant (IFA) (Sigma–Aldrich, Cat#: F5506) at a 1:1 ratio (*v/v*) and administered subcutaneously (*s.c*.) to the back of the neck. A week later (Day 7), BCG emulsified in IFA was injected into the left footpad (*i.f*.). After another week (Day 14), a booster immunization containing heat-killed *M. tuberculosis* without IFA was administered intraperitoneally (*i.p*.). The mice were sacrificed on Day 16, unless stated otherwise (Fig. 1a). While the second BCG dose (Day 7) was administered to the left footpad, all analyses were performed on the contralateral leg (right) to avoid any effects of local edema around the vaccination site and measure only the systemic effects of chronic inflammation.

### DSS-induced colitis – IBD

Mice were treated with 2.5% (*w/v*) DSS (Merck-Millipore, cat#: 160110) in the drinking water for 5 days. Weight was monitored for 14 days after DSS treatment, and the mice were sacrificed on Day 19. Colons were removed post-mortem and washed with saline before pictures were taken.

### Microcomputed tomography (microCT)

Right femora were fixed in PBS containing 4% paraformaldehyde (PFA) for 48 h at room temperature and stored at −20 °C. Bones were scanned using a Skyscan 1174 (Bruker) scanner. The scans were performed with a 7.6-μm voxel size. The mineralized tissues were segmented by a global thresholding procedure. Analyses of trabecular bone were performed in an area that was 0.6 mm below the distal growth plate. The length of the analyzed area of trabecular bone was 3 mm. Analyses of cortical bones were performed on the midshaft region of the bone.

### Histomorphometry

Mice were injected intraperitoneally with calcein (Sigma–Aldrich, Cat#: C0875) on Day 12 and Day 15 of the vaccination protocol to assess bone formation. Undecalcified femora were embedded in Technovit 9100 (Heraeus Kulzer, Cat#: 14655), and 8-µm longitudinal midfrontal plane sections were prepared for dynamic histomorphometric analysis as described before^40^. Dynamic histomorphometry was performed in the distal trabecular area, 0.6 mm from the growth plate into the femoral shaft. For the determination of bone resorption parameters, tibiae were fixed with 4% PFA for 48 h and decalcified with 10% EDTA for 2 weeks. After decalcification, the bones were embedded in paraffin and sectioned with a Leica microtome to a thickness of 5 μm. The sections were subjected to TRAP staining (Merck-Sigma–Aldrich, Cat#: 387A-1KT) for OC counting and imaged with an Eclipse Ci-L microscope, a DS-U3 imaging system with a DS-Fi2 camera and the NIS elements acquisition program (Nikon) at 20× magnification. For both dynamic and static analyses, ImageJ software was used.

### Serum collagen type I crosslinked C-telopeptide (CTX-I) measurement

Blood was collected from the heart of terminally anesthetized mice, and the serum was collected after clotting and stored at −80 °C. The CTX-I level was determined using a RatLaps CTX-I detection kit (Immuno-Diagnostic-Systems, Cat#: #AC-06F1) according to the manufacturer’s instructions.

### Flow cytometry and antibodies

Peripheral blood, spleen and BM cells were stained with antibodies against CD11b (Pacific Blue, M1/70), Ly6C (FITC, PE, or Alexa Fluor700; HK1.4), Ly6G (APC or biotin, 1A8), Thy1.2 (biotin or APC, 30-H12), B220 (biotin, RA3–6B2), Ter119 (biotin, TER-119), Gr1 (APC, RB6–8C5), CD115 (PE, AFS98), RANK (PE, R12–31), CD117 (PE, ACK2), FLT3 (PE, A2F10), Sca1 (PE/Cy7, D7), MHCII (FITC, M5/114.15.2), CD40 (Alexa Fluor 647, 3/23), CD80 (FITC, 16–10A1), CD86 (PE, PO3), CD11c (PE/Cy7, N418), F4/80 (PE or FITC, BM8), CD120a (APC, 55R-286), CD120b (REA228), CD3ε (APC, 145–2C11), CX_3_CR1 (PE, SA011F11), TGF-β (APC, TW7–16B4), or CD177 (Alexa Fluor 647, FAB8186R) and isotype controls (RTK2758 and MOPC-173), in combination with unlabeled anti-CD16/32 (clone 93). All antibodies were acquired from BioLegend, except those against MHCII (Tonbo), Sca1 (eBioscience), CD177 (R&D) and CD120b (Miltenyi). All cell surface staining was performed in PBS containing 1.5% fetal bovine serum (FBS) and 0.05% sodium azide (FACS buffer) on ice for 30 min, and then the cells were washed with FACS buffer. In the case of biotinylated antibodies, Alexa Fluor 647-conjugated streptavidin (Jackson) was used after any unbound primary antibodies were washed out. For intracellular staining of CD247, cells were fixed in 1% PFA in PBS for 20 min on ice, permeabilized with 0.1% saponin in PBS for 10 min at room temperature and stained with APC-conjugated anti-CD3ε and FITC-conjugated anti-CD247 (H146)^41^ in the presence of anti-CD16/32 in FACS buffer containing 0.1% saponin for 30 min on ice. Multicolor flow cytometry was performed using a Galios flow cytometer (Beckman-Coulter), and data were analyzed using FCS express v6 (DeNovo software).

### Isolation and sorting of Ly6C^hi^CD11b^hi/lo^ immature monocytes (iOCPs/hOCPs)

Monocytes were isolated from spleens and BM by magnetic separation (StemCell Technologies, Cat#: 19861A). The isolated monocytes were stained for Ly6C and CD11b, and Ly6C^hi^CD11b^hi^ (iOCPs) and Ly6C^hi^CD11b^lo^ (hOCPs) cells were sorted using a FACSARIA III (BD). The sorted cells were collected in MEM-α (Biological Industries) containing 15% FBS and 1.5% pen-strep solution. The samples were concentrated and counted twice using a TC20 automated cell counter (Bio Rad).

### OC cultures

Sorted cells (10^4^) were cultured in 96-well (flat-bottomed) plates in 200 µL MEM-α containing 10% FBS, 1% pen-strep solution, 20 ng·mL^−1^ RANKL (PeproTech, Cat#: 315–11–10), and 10% CMG14–12 conditioned medium as the source of M-CSF (equivalent to the bioactivity of 20 ng·mL^−1^ recombinant M-CSF), prepared as previously described^42^ (i.e., OC differentiation medium). Cultures were kept at 37 °C and 5% CO2 until TRAP staining. The medium was changed every 3 days. OCs were detected with a TRAP staining kit (Merck-Sigma–Aldrich, Cat#: 387A-1KT) as TRAP^+^ multinucleated cells (≥3 nuclei). Cultures were imaged using an Eclipse Ci-L microscope, a DS-U3 imaging system with a DS-Fi2 camera and the NIS elements acquisition program (Nikon) at 2× and 10× magnification, and the OC area was measured with Image-Pro Plus V6 (Media Cybernetics).

### Ex vivo T-cell suppression assay

Ninety-six-well (U-shaped) plates were precoated with anti-CD3ε (145–2C11) (3 μg·mL^−1^) and anti-CD28 (37.51) (3 μg·mL^−1^) antibodies in 100 µL of 0.1 mol·L^−1^ borate buffer (pH = 8.5) for 24 h at 4 °C. The wells were then blocked with borate buffer containing 1% FBS for 1 h at room temperature and washed with PBS three times. T cells were isolated from normal spleens by magnetic separation (StemCell Technologies, Cat#: 19851 A) and stained with CFSE (Thermo Fisher, Cat#: C34554) as previously described^20^. A total of 2 × 10^5^ CFSE-labeled T cells were seeded in RPMI medium (Gibco) supplemented with 8% FBS, 2 mmol·L^−1^ L-glutamine and 1% pen-strep solution (all from Biological Industries). The cultures were incubated at 37 °C and 5% CO_2_ for 1 h. Sorted iOCPs and hOCPs were seeded on top of the T cells at different T cell:MDSC ratios, and the cocultures were returned to the incubator for 80 h. Samples were harvested in cold FACS buffer and stained with antibodies against Thy1.2. The proliferation index was determined with an FCS-Express V6 proliferation analyzer (DeNovo software).

### Cell cycle staining

Surface staining of 2 × 10^6^ cells with biotinylated anti-Thy1.2, B220, Ly6G and Ter119 antibodies, in combination with anti-Ly6C-PE, anti-CD11b-FITC and anti-CD117-PE/Cy7 antibodies, was performed on ice for 30 min. The cells were stained with streptavidin-Alexa Fluor 647 for 15 min on ice. Then, the cells were fixed and permeabilized as in the CD247 staining protocol and stained with 10 μg·mL^−1^ DAPI (Merck-Sigma–Aldrich, Cat#: D9542) for 15 min on ice. Analysis was performed with an FCS-Express V6 multicycle DNA analyzer (DeNovo software).

### Immune effector differentiation assay

A total of 10^5^ sorted cells were cultured in 200 µL MEM-α containing 10% FBS, 1% pen–strep solution and 20 ng·mL^−1^ GM-CSF (PeproTech, Cat#: 315–03–10) or M-CSF (PeproTech, Cat#: 315–02–10). The cultures were kept at 37 °C and 5% CO2 for 3 days and then harvested by strong pipetting in cold 5 mmol·L^−1^ EDTA and 5 mmol·L^−1^ EGTA PBS buffer. Cells were stained for CD11c, MHCII and CD40 to assess DC and macrophage differentiation by flow cytometry.

### Pit formation assay

A total of 5 × 10^4^ sorted cells were cultured on Osteo assay surface plates (Corning, Cat# #3989) (96-well) in 200 µL of OC differentiation medium and incubated at 37 °C and 5% CO_2_ for 10 days until the OCs were exhausted in all cultures. The cultures were bleached with 6% sodium hypochlorite solution for 30 min, washed extensively with deionized water and air dried for 2 h. The cultures were imaged using an Eclipse Ci-L microscope, a DS-U3 imaging system with a DS-Fi2 camera and the NIS elements acquisition program (Nikon) at 4× and 20× magnification, and pit area was determined with Image-Pro Plus V6 (Media Cybernetics).

### Preparation of pure OC samples for proteome analysis

iOCPs and hOCPs were sorted from the BM of inflamed mice. A total of 3 × 10^5^ iOCPs or hOCPs were cultured in four technical replicates in 2 mL of MEM-α supplemented with 10% FBS, 1% pen–strep solution, 20 ng·mL^−1^ RANKL, and 25 ng·mL^−1^ recombinant M-CSF (PeproTech, Cat#: 315–02–10) in 12-well plates. The hOCP cultures were kept at 37 °C and 5% CO_2_ for 3 days, while the iOCP cultures were incubated for 5 days. Cultures were harvested using Accutase (Merck-Sigma–Aldrich, Cat#: B6964) for 40 min at 37 °C, and technical replicates were pooled together. The harvested cells were washed and resuspended in MEM-α supplemented with 10% FBS, 1% pen–strep solution and 10 μg·mL^−1^ Hoechst 33342 (Merck-Sigma–Aldrich, Cat#: B2261) for 30 min on ice for staining. The cells were washed; resuspended in MEM-α supplemented with 10% FBS, 1% pen–strep solution, 5 mmol·L^−1^ EDTA and 100 μg·mL^−1^ DNAse I (Merck-Sigma–Aldrich, Cat#: DN25); and filtered gently through a 100-µm nylon cell strainer. Mature OCs were sorted using an ARIAIII cell sorter (BD) as cells containing three or more nuclei. Sorting was performed at ~2 500 events/s, and cells were collected in MEM-α containing 10% FBS and 1% pen–strep solution. The sorted cells were concentrated, washed four times with 1 mL of ice-cold sterile PBS and pelleted for protein extraction. This protocol was performed as previously reported^31^.

### Lineage tracing of iOCP-OCs and hOCP-OCs

Control and inflamed WT mice received 10^6^ iOCPs or hOCPs sorted from the BM of inflamed *gfp* mice by intratibial injection into the right leg. The inflamed recipients received a single intrafootpad vaccination in the left leg 7 days before the implantation. Recipients were sacrificed 7 days after implantation. The right tibia and femur were fixed in 4% PFA–PBS for 24 h at 4 °C and equilibrated in 15% and 30% sucrose PBS reciprocally, each overnight; no decalcification was performed. Bones were embedded in OCT and frozen at −80 °C. Consecutive 7-µm sections were cut using a Leica cryostat as previously described^43^. Briefly, 7-µm cryosections were made on CryoJane tape windows (Leica, Cat#: 39475214) and then transferred to carrier slides freshly coated with a thin layer of Norland optical adhesive #63 (Norland, USA) by UV (375 nm) illumination for 30 s. Consecutive sections were alternately stained for TRAP and GFP to detect donor-derived OCs. TRAP staining was performed using a leukocyte phosphatase detection kit (Merck-Sigma–Aldrich, Cat#: 387 A) according to the manufacturer’s instructions. For GFP detection, slides were quenched in 3% H_2_O_2_ for 10 min, followed by 1 h of blocking in 5% normal horse serum in CAS-block (Thermo-Fisher, Cat#: 008120) containing 0.05% Tween-20. An anti-GFP antibody (Abcam, Cat#: ab6673) was used at a 1:500 dilution in PBS containing 0.05% Tween-20 and 5% horse serum for 1.5 h. Peroxidase-conjugated horse anti-goat IgG (Vector labs, Cat#: MP-7405) was added for 0.5 h, and detection was performed using DAB (Abcam, Cat#: ab64238). Hematoxylin was used for counterstaining. Mounted samples were imaged with an Olympus microscope and a DP74 camera (Olympus) at 6.3× magnification.

### Quantitative PCR

Total RNA was extracted from snap-frozen tibiae by homogenization in Tri-reagent (Sigma–Aldrich). cDNA was prepared with m-MLV reverse transcriptase (Quanta, Cat#: 95047–100). Quantitative real-time PCR (ABI 7900) was performed using SYBR green (Thermo Fisher Scientific, Cat#: 11744500). The primer sequences are listed in Supplementary Table 1, and each primer pair was situated across exon-exon junctions. *Murine* ubiquitin C was used as a housekeeping gene in all experiments. All results are presented as the relative quantity.

### Protein extraction

Pellets of sorted cells were lysed in 2% SDS in 25 mmol·L^−1^ Tris (pH = 8) supplemented with 10 mmol·L^−1^ DTT, 1 mmol·L^−1^ PMSF and 1 mmol·L^−1^ EDTA on ice. The lysates were sonicated to 20 kJ three times in ice-cold water. Samples for western blot analyses were mixed with loading dye, incubated for 10 min at 95 °C and stored at −20 °C. Lysates for proteomic analyses were incubated at 50 °C for 10 min and stored at −20 °C.

### Mass spectrometry (MS) sample preparation

Protein concentrations were determined with Coomassie dot blotting using known BSA standards. Reduction/alkylation was performed for 5 min at 50 °C with 10 mmol·L^−1^ DTT and for 25 minutes at 45 °C with 40 mmol·L^−1^ IAA in the dark, and the reaction was quenched with 20 mmol·L^−1^ DTT at RT.

Next, for OCPs, automated SP3 cleanup was performed as previously described^44^. In summary, the Bravo system was programmed to process four replicates of the four experimental conditions simultaneously, carrying out all handling steps, including aliquoting of magnetic beads, protein clean-up by SP3, protein digestion, and peptide recovery in a 96-well plate. A starting sample volume of 20 μL containing 7.5 μg of sonicated lysates was combined with 5-μL aliquots of a suspension of washed magnetic beads (50 μg·μL^−1^). Then, 25 μL of 100% acetonitrile (ACN) was added to each sample, followed by 18 min of incubation off the magnetic rack with cycles of agitation. The sample plate was incubated on the magnetic rack for an additional 5 min. Next, the beads were washed two times with 200 μL of 80% EtOH and one time with 100% ACN. Upon removal of residual washing solvents, the beads were resuspended in 35 μL of 100 mmol·L^−1^ ammonium bicarbonate and 5 μL of 0.05 μg·μL^−1^ trypsin. Next, the plate was shaken at 1 500 r·min^−1^ for 60 s before being transferred to the heating deck position for overnight digestion at 37 °C. Digestion was stopped by manually adding 2 μL of 10% trifluoroacetic acid (TFA). The solution was sonicated in a water bath for 5 min and incubated on a magnetic rack for 2 min. Finally, the peptide-containing supernatants were transferred into a new 96-well plate. Peptide solutions (~2.5 µg, 15 μL) were analyzed as single-shot MS injections, and 5 μg from each sample was combined and fractionated off-line using reversed-phase high-pH fractionation. For OCP-OCs, SP3 cleanup was performed manually. Two microliters of washed SP3 magnetic beads (50 μg·μL^−1^) was combined with 2 μg of protein lysate in 25 μL. ACN was added to a final concentration of 60%, and the beads were washed twice with 80% EtOH. The proteins were eluted in 18 μL of 50 mmol·L^−1^ ammonium bicarbonate containing 40 ng of Trypsin/Lys-C mixture and digested for 16 h. The reaction was stopped by the addition of 2 μL of 10% formic acid. One microgram of peptide solution was analyzed as single-shot MS injections in two technical replicates.

### Chromatography

For LCMS analysis, peptides were separated using an Easy NanoLC 1200 fitted with a trapping (Acclaim PepMap C18, 5 μm, 100 Å, 100 μm × 2 cm) and an analytical column (Waters nanoEase MZ Peptide BEH C18, 130 Å, 1.7 µm, 75 µm × 25 cm). Solvent A was 0.1% (*v*/*v*) formic acid (FA) in ddH_2_O, and solvent B was 80% ACN and 0.1% (*v*/*v*) FA in ddH_2_O. Samples were loaded onto the trapping column with a constant flow of solvent A at a maximum pressure of 800 bar. Peptides were eluted at a constant flow of 0.3 μL·min^−1^ and temperature of 55 °C and maintained using a HotSleevePlus column oven (Analytical Sales and Services). During elution, the percentage of solvent B was increased linearly from 3 to 8% in 4 min, from 8% to 10% in 2 min, from 10% to 32% in 68 min, from 32% to 50% in 12 min, and from 50% to 100% in 1 min. Finally, the gradient was finished with 7 min in solvent B, followed by 11 min at 97% solvent A.

Fractionation of the combined OCPs occurred on an Agilent Infinity 1260 LC system (Agilent) using a Phenomenex Gemini 3 μmol·L^−1^ C18, 100 × 1 mm column (Phenomonex). Buffer A was 20 mmol·L^−1^ NH_4_COOH, and buffer B was 100% can. The following gradient was used: 0–2 min, 0% B; 2–60 min, linear gradient to 65% B; 61–62 min, linear gradient to 85% B; 62–67 min, 85% B; and 67–85 min, 0% B. The eluates were collected in 40 fractions and combined in 16 fractions. The 16 fractions were dried with a SpeedVac, and the peptides were resuspended and cleaned using an Oasis PRiME HLB μElution Plate (Waters) and finally resuspended in 15 μL 0.5% TFA.

### Proteomic data acquisition

Peptides were introduced into mass spectrometers via a Pico-Tip Emitter 360 μm OD × 20 μm ID; 10 μm tip (New Objective). The capillary temperature was set at 275 °C.

OCP samples were analyzed on a Q-Exactive HF Orbitrap mass spectrometer (Thermo Fisher Scientific) with a spray voltage of 2 kV. Full-scan MS spectra with a mass range of *m/z* 350 to 1 500 were acquired with the Orbitrap at a resolution of 60 000 FWHM. The filling time was set to a maximum of 50 ms with an automatic gain control target of 3 × 10^6 ^ions. The top 20 most abundant ions per full scan were selected for MS^2^ acquisition. Dynamic exclusion was set for 25 s. Isotopes, unassigned charges, and charges of 1 and >8 were excluded. For MS^2^ scans, the normalized collision energy was set to 26, and the resolution was set to 15 000 FWHM with automatic gain control of 1 × 10^5 ^ions and a maximum fill time of 50 ms. The isolation window was set to m/z 2, with a fixed first mass of *m*/*z* 110.

OCP-OC samples were analyzed on a Fusion Orbitrap mass spectrometer (Thermo Fisher Scientific) with a spray voltage of 2.5 kV. Full-scan MS spectra with a mass range of *m/z* 375 to 1 500 were acquired with the Orbitrap at a resolution of 60 000 FWHM. The filling time was set to a maximum of 50 ms with an automatic gain control target of 250%. Master scans were performed every 3 s. Ions with intensities above 5 × 10^3^ were selected for MS^2^ acquisition and detection with an ion trap. Dynamic exclusion was set for 20 s. Isotopes, unassigned charges, and charges of 1 and >4 were excluded. For MS^2^ scans, the normalized collision energy was set to 33, with standard automatic gain control and a maximum fill time of 50 ms. The isolation window was set to *m*/*z* 1.6.

### Proteomic data processing

Raw files were processed using MaxQuant (version 1.6.2.6)^45^. Technical replicates were combined using identical sample names. A search was performed against the mouse UniProt database (20180716_Uniprot_mus-musculus_canonical_reviewed) using the Andromeda search engine with the following search criteria: enzyme was set to trypsin/P with up to two missed cleavages. Carbamidomethylation (C) and oxidation (M)/acetylation (protein N-term) were selected as fixed and variable modifications, respectively. The first and second search peptide tolerances were set to 20 and 4.5 ppm, respectively. Protein quantification was performed using the label-free quantification (LFQ) algorithm of MaxQuant. MS^2^ spectra were not required for the LFQ comparison. In addition, intensity-based absolute quantification (iBAQ) intensities were calculated with log fit enabled. Identification transfer between runs via the matching between runs algorithm was allowed. Peptide and protein hits were filtered at a false discovery rate of 1%, with a minimal peptide length of 7 amino acids. The reversed sequences of the target database were used as a decoy database. All remaining settings were default MaxQuant settings. LFQ values were extracted from the protein groups table and log_10_-transformed for further analysis. Proteins that were only identified by a modification site, contaminants, and the reversed sequences were removed from the dataset. All further analyses were performed using either Perseus (version 1.6.1.3)^46^ or R software (version 3.3.3)^47^ packages available through BioConductor^48^. In particular, differential expression analysis of the samples was performed using Limma moderated t-statistics (R package version 3.30.1)^49^. The Benjamini–Hochberg correction was used to calculate adjusted *P values*.

Only protein groups with a valid measurement in two out of four replicates were subjected to the Limma algorithm. LFQ values that were missing in all replicates for a specific experimental condition were imputed using values obtained from a random distribution having a mean and an SD equal to the minimum LFQ value measured in the experiment. The random distribution was obtained with the urnorm function of the Runuran package (version 0.24) with the lower boundary set to 0.

Principal component (PC) analysis was performed using the prcomp function. GO term enrichment analyses were performed using the enrichGO function of the clusterProfiler package (version 3.2.14). The Benjamini–Hochberg FDR was set at 0.05 for the enrichment assay. For OCPs, the analysis was performed separately for PCs 1–4 on the top or bottom 5% of proteins sorted according to respective PC loadings. For OCP-OCs, either positively or negatively differentially expressed proteins (Limma, Benjamini–Hochberg FDR = 0.05) were used. The entire list of proteins detected in each dataset was used as the background.

All *R* scripts used in data analyses and figure generation are available upon request.

### Western blot analysis

One microgram of total protein was loaded onto a 12% polyacrylamide gel. The proteins were blotted onto a nitrocellulose membrane. For the detection of RAGE and PP2Ac, membranes were blocked with 5% BSA in TBST and probed with an anti-RAGE (Abcam, Cat#: Ab3611) or anti-PP2Ac (R&D, Cat#: AF1653) antibody, followed by incubation with peroxidase-conjugated donkey anti-rabbit IgG (BioLegend, Cat#: 406401). For the detection of S100A8/A9, membranes were blocked in 10% skim milk in PBST and probed with goat polyclonal antibodies against S100A8 (R&D, Cat#: AF3059) and S100A9 (R&D, Cat#: AF2065), followed by incubation with peroxidase-conjugated rabbit anti-goat IgG (Jackson ImmunoResearch Labs, Cat#: 305–035–045). Chemiluminescence detection was performed with Clarity western ECL (Bio-Rad, Cat#: 170–5061), blots were captured with a ChemiDoc XRS + molecular imager (Bio-Rad), and densitometry was evaluated with Image Lab (Bio-Rad).

### Statistical analysis

All data are presented as the median (line), 25th–75th percentile (box) and range (whiskers). An exact two-tailed Mann–Whitney test was performed for comparisons of two groups. To compare multiple groups to a single control, an exact Kruskal–Wallis test followed by Dunn’s test was performed. For all quadruple comparisons (*i.e*., inflamed and control WT/*tnf-α*^−/−^, inflamed and control iOCP/hOCP, etc.), the effect of each experimental condition on the four experimental groups was examined separately using an exact Kruskal–Wallis test; those few that did not produce significant results were not analyzed further. All other effects were then tested separately using exact two-tailed Mann–Whitney tests, and multiple comparisons within each experiment were adjusted using the Bonferroni correction. To compare iOCPs to hOCPs, which were dependent variables as they came from the same mouse, an extra penalty and Holm correction were used to adjust for multiple comparisons. *P* values less than 0.05 were considered significant.

## Supplementary information

Figures s1–s6

Supplementary tables

## Data Availability

The mass spectrometry proteomic data have been deposited in the ProteomeXchange Consortium^50^ via the PRIDE^51^ partner repository with the dataset identifier PXD031791.
