# Supplementary material for: Specific inflammatory osteoclast precursors induced during chronic inflammation give rise to highly active osteoclasts associated with inflammatory bone loss
Source: Bone Res. 2022 Apr 8;10:36. doi: 10.1038/s41413-022-00206-z (PMC8993801; doi:10.1038/s41413-022-00206-z)
Supplement: Supplementary file 1 — Figures s1–s6 [file 41413_2022_206_MOESM1_ESM.pdf]

Supplementary Figures

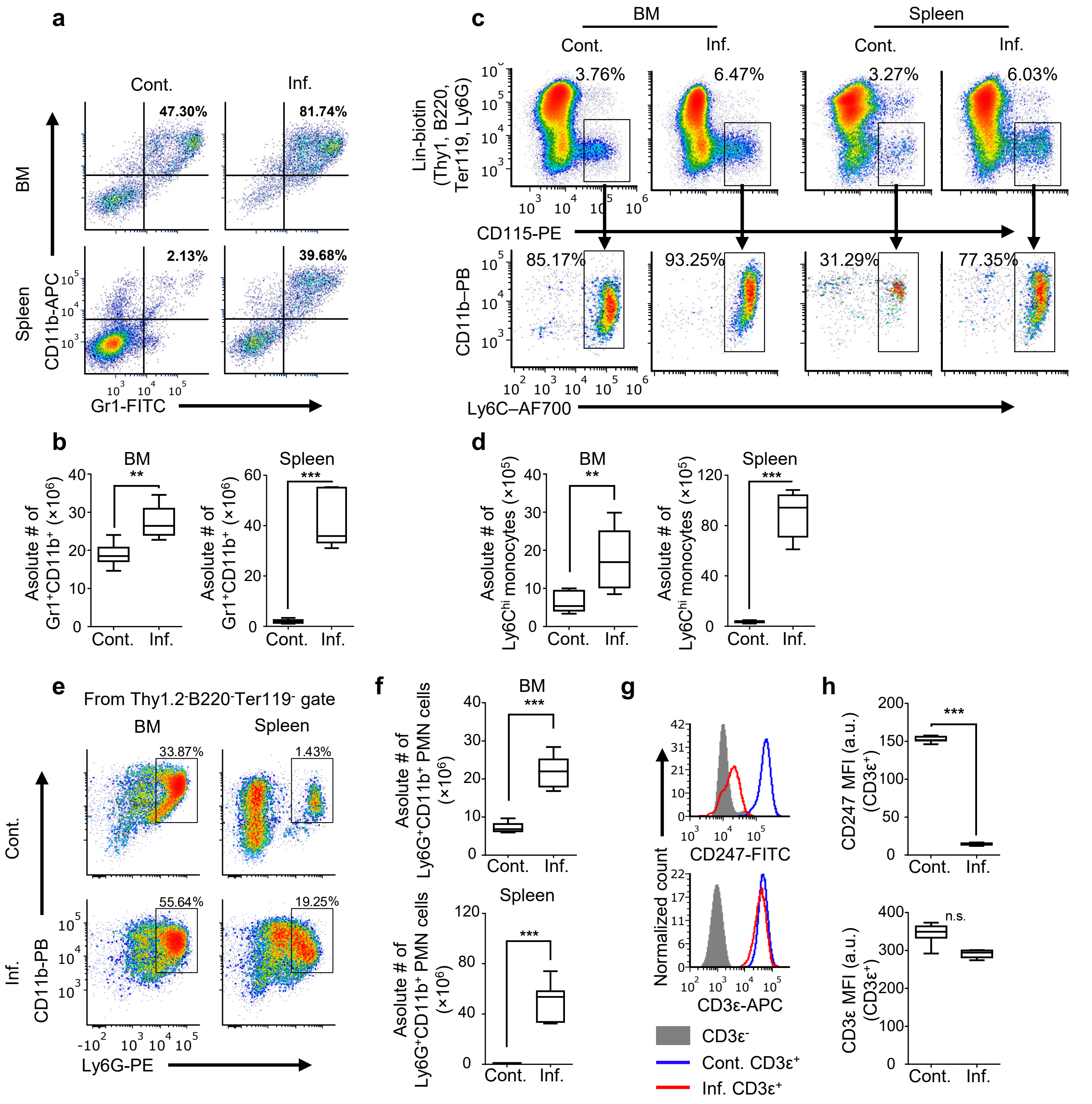

Fig. S1 Continuous immune stimulation elicits a general chronic inflammation. **a.** Representative plots of the general Gr1$^+$CD11b$^+$ MDSC population in the BM and spleen of control and inflamed mice. **b.** The absolute number of MDSCs in the BM and spleen of inflamed *vs.* control mice. **c.** The distribution of Lin$^-$ (Thy1$^-$B220$^-$Ter119$^-$Ly6G$^-$) CD115$^+$Ly6C^{hi}CD11b$^+$ (Ly6C^{hi} monocytes) in the BM and the spleen. **d.** Absolute number of Ly6C^{hi} monocytes in the BM and spleen. **e.** The distribution of Ly6G$^+$CD11b$^+$ PMN-MDSC in the BM and spleen of control and inflamed mice. **f.** Absolute number of Ly6G$^+$CD11b$^+$ PMN-MDSCs in the BM and spleen. **g.** Representative histograms of CD247 and CD3ε expression by splenic T-cells. **h.** Quantitaion of CD247 and CD3ε expression in control and inflamed Splenic T-cells, presented as mean fluorescence intensity (MFI) in arbitrary units (a.u.). Line: median, box: 25th-75th percentile, whiskers: range. Control N=8, inflamed N=8. ** P<0.01, *** P<0.001 (Mann-Whitney test).

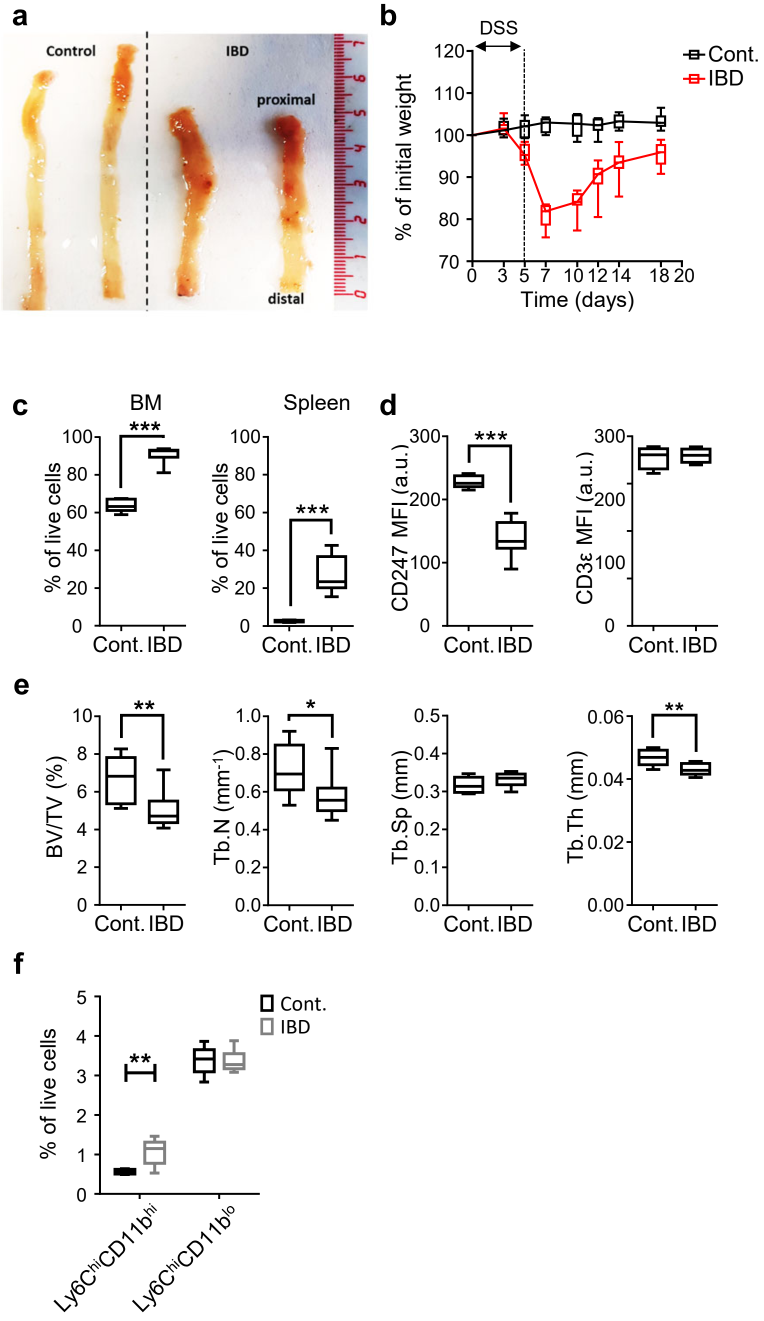

Fig. S2 Chronic inflammation in IBD leads to bone loss and iOCP but not hOCP accumulation in the BM. **a.** Representative colons of control and IBD mice at endpoint (scale in cm). **b.** Weight change during and after a 5-day DSS treatment, mice were sacrificed at day 19. **c.** The fraction of Gr1^{+}CD11b^{+} MDSCs in the BM and the spleen. **d.** CD247 and CD3ε expression in splenic T-cells. **e.** Trabecular measurements: BV/TV, Tb.N, Tb.Sp and Tb.Th. **f.** The fraction of Ly6C^{hi}CD11b^{hi} and Ly6C^{hi}CD11b^{lo} in the BM of control *vs.* IBD mice. Control N=8, IBD N=8, representative results of 2 independent experiments. Line: median, box: 25th-75th percentile, whiskers: range. * P<0.05, ** P<0.01, *** P<0.001 (Mann-Whitney test and Holm multiplicity correction).

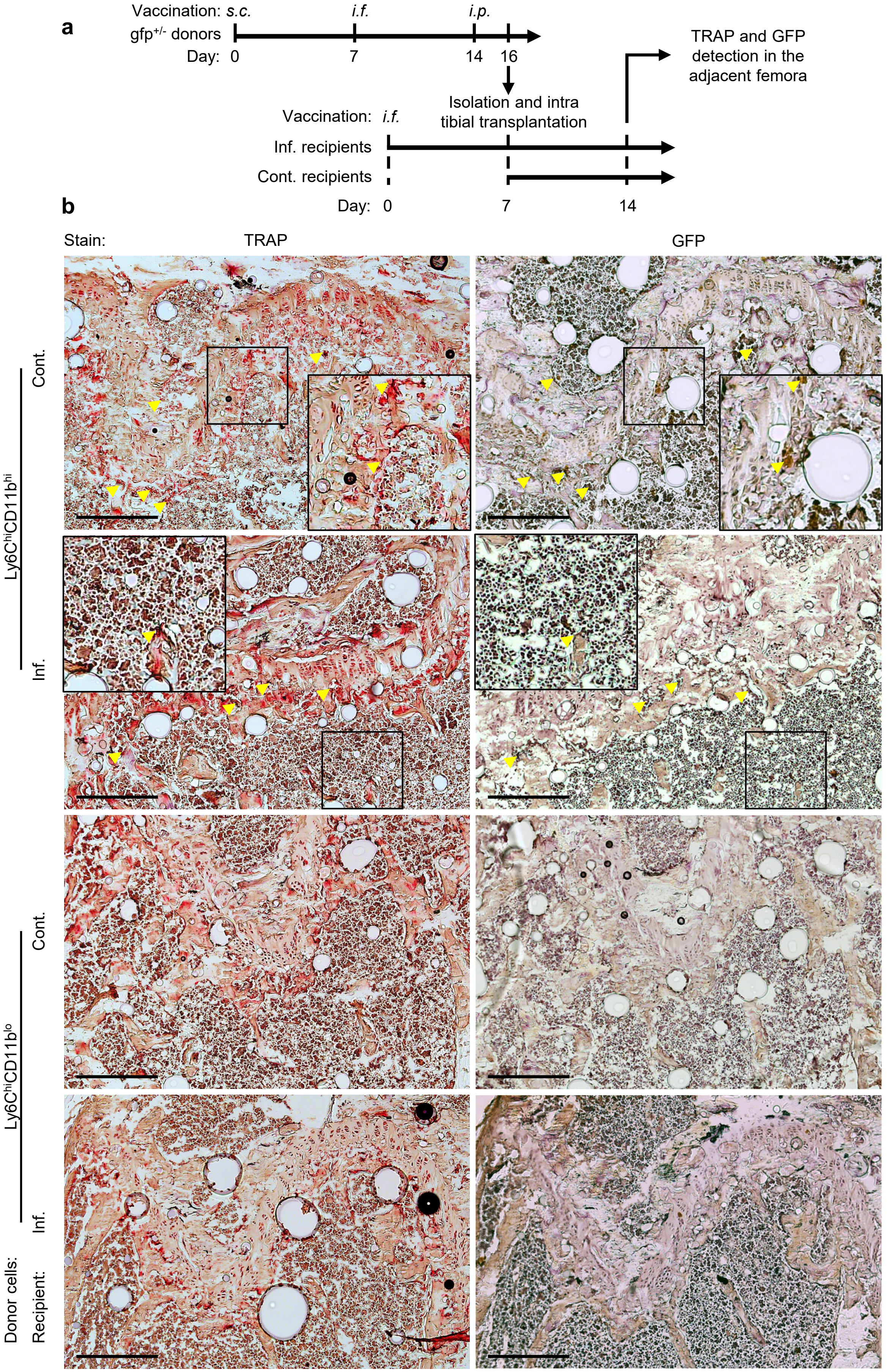

Fig. S3 Ly6C^{hi}CD11b^{hi} and Ly6C^{hi}CD11b^{lo} give rise to OCs *in vivo*. **a.** Sorted 10^6 Ly6C^{hi}CD11b^{hi} or Ly6C^{hi}CD11b^{lo} from BM of inflamed *gfp*$^{+/-}$ donor mice were injected to the right tibiae of inf. WT recipient mice 7 days after a single *i.f.* vaccination (left foot pad), or to sex/age matched cont. mice. Recipient mice were sacrificed 7 days after the transplantation. **b.** TRAP staining (left panel) and GFP immunohistochemistry (right panel) of 7µm consecutive sections from the right femora of the recipient mice. Black frames are digitally enlarged, locations positive for both TRAP and GFP are marked by yellow arrows. Scale bar 400µm.

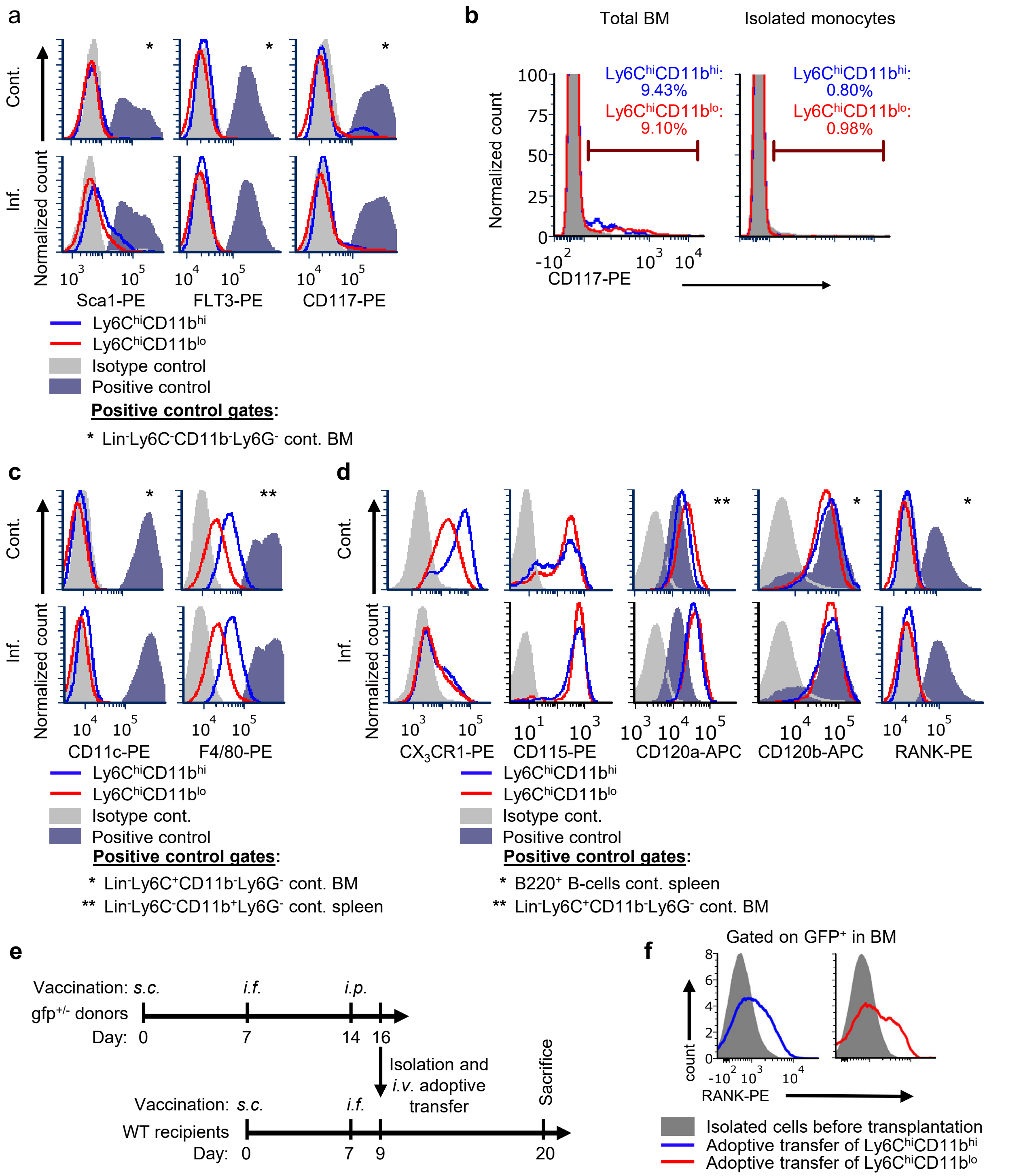

Fig. S4 Expression of known OCP surface markers in Ly6C^{hi}CD11b^{hi} and Ly6C^{hi}CD11b^{lo}. A phenotypical screen of surface markers expressed by Ly6C^{hi}CD11b^{hi} and Ly6C^{hi}CD11b^{lo} in the BM of inflamed and control mice. Isotype control is depicted as gray filled area and positively stained internal controls are depicted as blue-gray filled areas (gates and samples of positive controls are indicated by asterisks). **a.** Stem cell and myeloid progenitor markers Sca1, FLT3 and CD117. **b.** CD117^{+} cells are depleted from isolated monocytes (used in all the following *ex vivo* experiments). **c.** Myeloid differentiation markers CD11c and F4/80. **d.** Expression of the known OCP markers CX$_3$CR1, CD115 and the two TNF-α receptors 1 and 2 (CD120a and CD120b, respectively) and RANK, by Ly6C^{hi}CD11b^{hi} and Ly6C^{hi}CD11b^{lo} in control and inflamed mice. **e.** Sorted 2.5×10^{6} Ly6C^{hi}CD11b^{hi} or Ly6C^{hi}CD11b^{lo} from BM of inflamed *gfp*$^{+/-}$ donor mice were injected *i.v.* to inflamed WT recipient mice on day 9 of the vaccination treatment, recipient mice were sacrificed on day 20. **f.** RANK expression in Ly6C^{hi}CD11b^{hi} and Ly6C^{hi}CD11b^{lo}, sorted from *gfp*$^{+/-}$ mice, before adoptive transfer and in the BM of recipient mice 11 days after adoptive transfer.

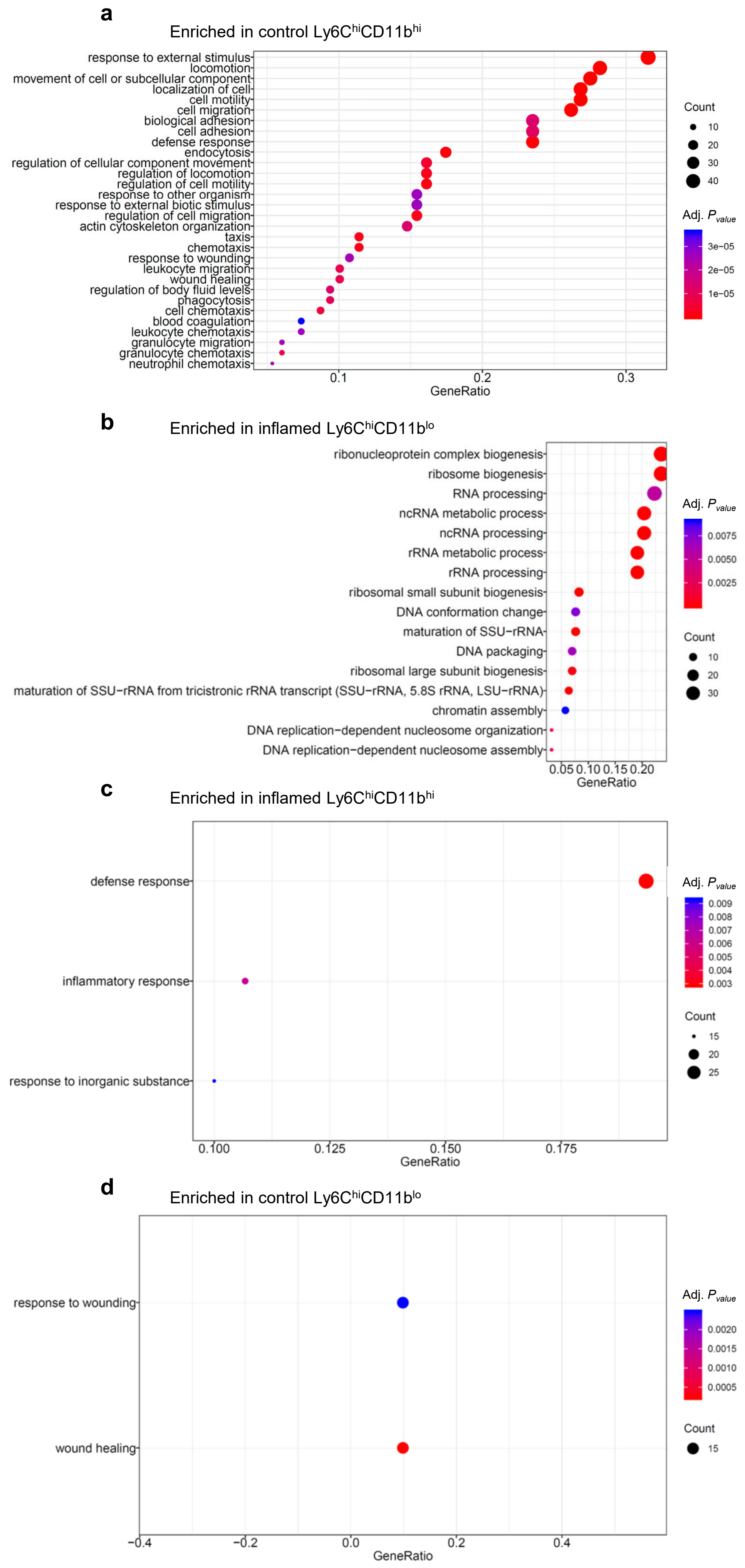

Fig. S5 Enriched biological process categories for Ly6C^{hi}CD11b^{hi} and Ly6C^{hi}CD11b^{lo}. **a.** Enriched in control Ly6C^{hi}CD11b^{hi} (PC1 bottom 5% loading scores). **b**. Enriched in inflamed Ly6C^{hi}CD11b^{lo} (PC1 top 5% loading scores). **c.** Enriched in inflamed Ly6C^{hi}CD11b^{hi} (PC2 bottom 5% loading scores). **d.** Enriched in control Ly6C^{hi}CD11b^{lo} (PC3 top 5% loading scores). Benjamini-Hochberg FDR was set to 0.05. Uniprot biological process GO terms were used. Symbol size represents the number of proteins mapped to each category (count). Adjusted *P-value* is color coded.

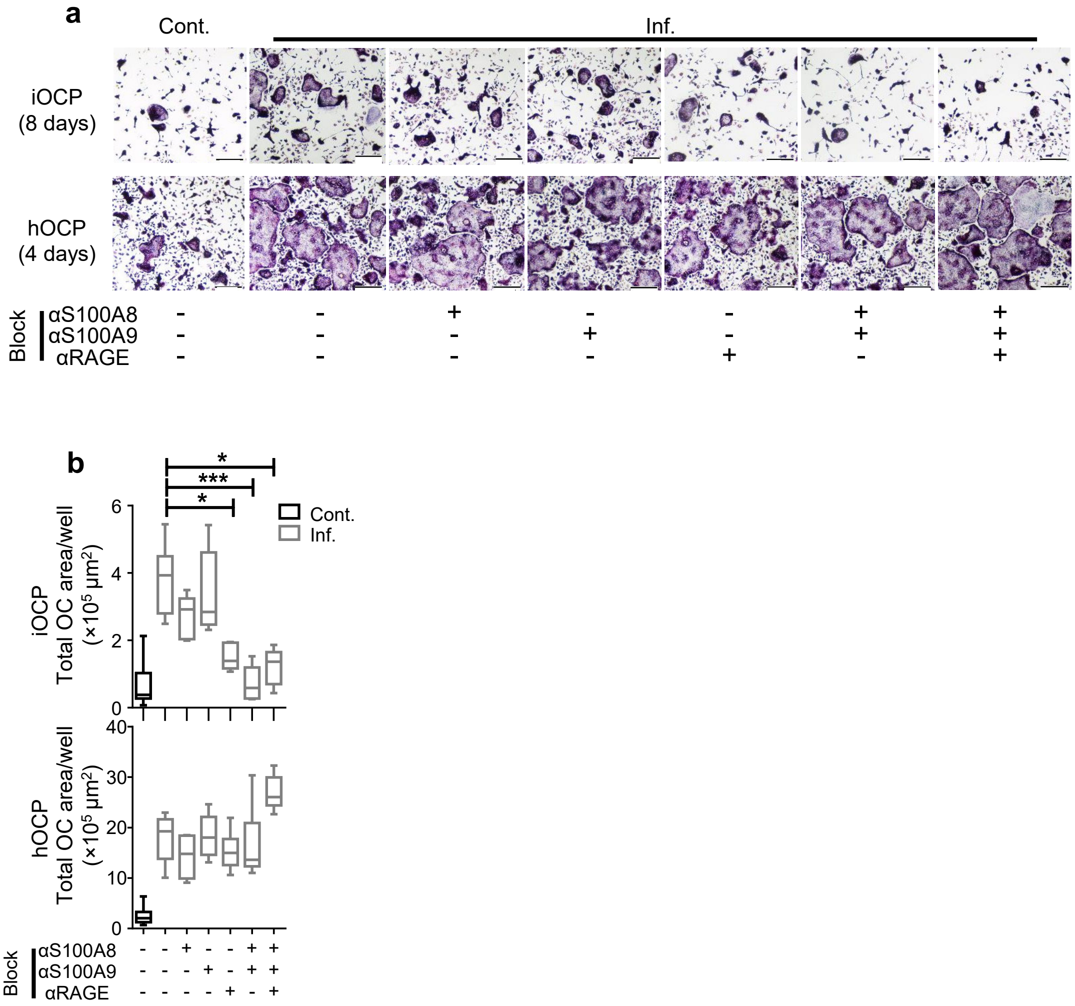

Fig. S6 The RAGE-S100A8/A9 axis controls OC differentiation from iOCPs but not from hOCPs. **a.** Sorted 10^4 iOCPs and hOCPs from BM of inflamed mice were cultured in OC differentiation medium for 8 and 4 days, respectively with blocking antibodies for S100A8, S100A9 and RAGE, in combinations as indicated (200μm bar). **b.** OC area quantitation. iOCPs and hOCPs from BM of control mice are presented in black. N=5 for each group, representative results of 2 independent experiments. Line: median, box: 25th-75th percentile, whiskers: range. * P<0.05, ** P<0.01, *** P<0.001 (Kruskal-Wallis test and Dunn post test).
