# Supplementary material for: Specific inflammatory osteoclast precursors induced during chronic inflammation give rise to highly active osteoclasts associated with inflammatory bone loss
Source: Bone Res. 2022 Apr 8;10:36. doi: 10.1038/s41413-022-00206-z (PMC8993801; doi:10.1038/s41413-022-00206-z)
Supplement: Supplementary file 2 — Supplementary tables [file 41413_2022_206_MOESM2_ESM.docx]

|  | **Description** | **Measurement** | **Cont.** | **Inf.** | **Δ (%)** | ***P_value_*** |
| --- | --- | --- | --- | --- | --- | --- |
| **Trabecular bone** | **Bone volume fraction** | **BV/TV(%) *** | **6.322 ± 1.412** | **4.143 ± 1.077** | **-34.472** | **0.0262** |
|  | Trabecular thickness | Tb.Th (mm) | 0.052 ± 0.004 | 0.052 ± 0.002 | 0.466 | 0.7104 |
|  | **Trabecular number** | **Tb.N (mm^-1^) *** | **1.223 ± 0.280** | **0.800 ± 0.226** | **-34.580** | **0.0111** |
|  | **Trabecular separation** | **Tb.Sp (mm) *** | **0.294 ± 0.037** | **0.359 ± 0.062** | **22.185** | **0.0140** |
|  | OC number/bone perimeter | Oc.N/BPm (mm^-1^) | 8.929 ± 3.061 | 7.450 ± 2.076 | -16.560 | 0.4225 |
|  | OC perimeter/bone perimeter | Oc.Pm/BPm (%) | 12.071 ± 3.908 | 10.794 ± 3.319 | -10.578 | 0.7308 |
|  | Mineral apposition rate | MAR (µm/day) | 3.852 ± 0.820 | 4.118 ± 0.692 | 6.904 | 0.4848 |
|  | Bone formation rate | BFR (µm^3^/µm^2^/day) | 1.840 ± 0.434 | 2.036 ± 0.302 | 10.653 | 0.3939 |
| **Cortical bone** | **Cross-sectional cortical area** | **Ct.Ar (mm^2^) *** | **0.606 ± 0.026** | **0.537 ± 0.026** | **-11.388** | **0.0006** |
|  | **Cross-sectional cortical area fraction** | **Ct.Ar/Tt.Ar (%) *** | **32.392 ± 1.001** | **28.927 ± 1.187** | **-10.696** | **0.0006** |
|  | OC number/bone perimeter | Oc.N/BPm (mm^-1^) | 0.004 ± 0.001 | 0.005 ± 0.001 | 34.344 | 0.3429 |
|  | **OC perimeter/bone perimeter** | **Oc.Pm/BPm (%) *** | **0.151 ± 0.027** | **0.260 ± 0.061** | **72.097** | **0.0286** |

**Table S1** All WT bone analyses. MicroCT scans and histomorphometry of the distal femur of control and inflamed mice. Measurements are presented as the mean ± standard deviation and the % difference between the means (Δ) (inflamed - control). All microCT scans and histological evaluations were performed on the right femur. Representative results for 3 independent experiments; control N=7, inflamed N=7. *P_values_* < 0.05 are bolded and marked by asterisks (Mann–Whitney test and Holm multiplicity correction).

**Table S2** Primer list

| **Target** | **Forward (5’ – 3’)** | **Reverse (5’ – 3’)** |
| --- | --- | --- |
| *Tnf-α* | GCCACCACGCTCTTCTGTCTAC | GGGTCTGGGCCATAGAACTGAT |
| *M-csf* | ATGCCAGATTGCCTTTGAAT | CATGGAAAGTTCGGACACAG |
| *Rankl* | CATCGGGTTCCCATAAAGTC | CTTGGGATTTTGATGCTGGT |
| *Opg* | ACCAAAGTGAATGCCGAGAG | TCTCCATCAAGGCAAGAAGC |
| *Rage* | AACGGGATCTTTCACAGAGACG | GGCTGCTTGGAATAGACACTC |
| *S100a8* | GGAAATCACCATGCCCTCTACAA | ATGCCACACCCACTTTTATCACC |
| *S100a9* | GGAGCGCAGCATAACCACCATC | GCCATCAGCATCATACACTCCTCA |
| *mUbc* | CAGCCGTATATCTTCCCAGACT | CTCAGAGGGATGCCAGTAATCTA |
